# Human adipose mesenchymal stem cell-derived exosomal-miRNAs are critical factors for inducing anti-proliferation signalling to A2780 and SKOV-3 ovarian cancer cells

**DOI:** 10.1038/srep38498

**Published:** 2016-12-08

**Authors:** Abu Musa Md Talimur Reza, Yun-Jung Choi, Hideyo Yasuda, Jin-Hoi Kim

**Affiliations:** 1 Department of Stem Cell and Regenerative Biology, Humanized Pig Research Centre (SRC), Konkuk University, Seoul 143-701, Republic of Korea

## Abstract

An enigmatic question exists concerning the pro- or anti-cancer status of mesenchymal stem cells (MSCs). Despite growing interest, this question remains unanswered, and the debate became intensified with new evidences backing each side. Here, we showed that human adipose MSC (hAMSC)-derived conditioned medium (CM) exhibited inhibitory effects on A2780 human ovarian cancer cells by blocking the cell cycle, and activating mitochondria-mediated apoptosis signalling. Explicitly, we demonstrated that exosomes, an important biological component of hAMSC-CM, could restrain proliferation, wound-repair and colony formation ability of A2780 and SKOV-3 cancer cells. Furthermore, hAMSC-CM-derived exosomes induced apoptosis signalling by upregulating different pro-apoptotic signalling molecules, such as BAX, CASP9, and CASP3, as well as downregulating the anti-apoptotic protein BCL2. More specifically, cancer cells exhibited reduced viability following fresh or protease-digested exosome treatment; however, treatment with RNase-digested exosomes could not inhibit the proliferation of cancer cells. Additionally, sequencing of exosomal RNAs revealed a rich population of microRNAs (miRNAs), which exhibit anti-cancer activities by targeting different molecules associated with cancer survival. Our findings indicated that exosomal miRNAs are important players involved in the inhibitory influence of hAMSC-CM towards ovarian cancer cells. Therefore, we believe that these comprehensive results will provide advances concerning ovarian cancer research and treatment.

Different organs including ovaries are surrounded and supported by adipose fat-pad, which provide physical, as well as mechanical supports and play important roles during organogenesis, morphogenesis, disease-progression of respective organs^1^. Being an important composite of adipose-stromal cells, adipose mesenchymal stem cells possibly have regulatory part in different cancers, such as ovarian cancer. However, relationships between mesenchymal stem cells (MSCs) and cancer cells are a mystery, owing to insufficient evidence concerning both the stimulatory and inhibitory roles of MSCs on cancer cells^2^. While there is debate about the customary roles of MSCs, their involvement in cancer biology is undoubtedly clear. MSCs potentially support tumour development through immune suppression, epithelial-to-mesenchymal transition^1^, angiogenesis, and serving as cancer stromal cells^3^. In contrast, MSCs also suppress cancer by downregulating cancer survival-signalling pathways involving WNT/β-catenin and/or AKT^4^. There is a need to investigate the mechanisms underlying the contradictory roles associated with MSCs in cancer biology. Cytokines and soluble factors secreted by MSCs have been thoroughly scrutinized, with most reports concluding that MSC-secreted cytokines and soluble factors exhibit stimulatory effects related to cancer progression^2^,^5^.

Exosomes are types of membrane-bound micro-vesicles 30 nm to 200 nm in diameter, found in bio-fluids and contain many important components, including RNA, proteins, DNA, and lipids, and serve as efficient vehicles for cancer-stromal communication^6^. Exosomes are secreted by all cells and, despite their ability to be incorporated into neighbouring cells, have been only marginally investigated. Specifically, cell-secreted microRNAs (miRNAs; 18–22 nucleotides) are predominantly carried by exosomes and have been studied in recent years for their roles in post-transcriptional regulation of gene expression through mRNA silencing^7^. Therefore, understanding the functions of the MSC-derived secretome (particularly exosomes) in cancer is critical to elucidating the cross-talk between MSCs and cancer cell biology.

In this study, we hypothesized that human adipose-derived MSC (hAMSC)-secreted biological component (cytokines, miRNAs and others) might have important influence on the regulation of ovarian cancers. Hence, we investigated the influence of hAMSC-secreted molecules on different ovarian cancer cells. Our results showed that hAMSC-conditioned medium (hAMSC-CM)-derived exosomes treatment inhibited the proliferation and growth of A2780 and SKOV-3 ovarian cancer cells. More precisely, cancer cells exhibited reduced viability, wound healing, and colony formation following fresh or protease-digested exosome treatment; however, treatment with RNase-digested exosomes could not inhibit the proliferation of A2780 and SKOV-3 cancer cells. Furthermore, sequencing of exosomal RNAs revealed a rich population of miRNAs, with many reported to exhibit anti-cancer properties through targeting different cancer-survival pathways. Our findings indicated that exosomes (particularly exosomal miRNAs) may be one explanation for the anti-proliferative effects exhibited by hAMSC-CM, and that the relationship between MSCs and cancer could be partially explained by exosome-related activity. These results provided valuable insights into the diversity, enrichment, and function of all miRNAs derived from hAMSC-secreted exosomes.

## Results

### hAMSC-CM treatment reduced proliferation of A2780 ovarian cancer cells

**Figure 1:**
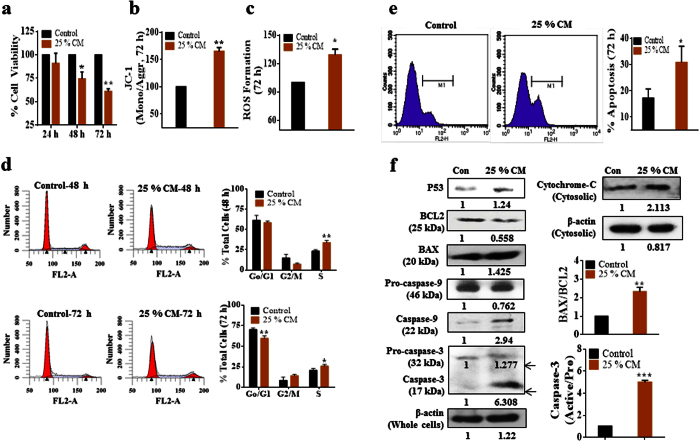
hAMSC-CM inhibited the growth and proliferation of A2780 ovarian cancer cells *in vitro*. (**a**) Bar diagram showing the percentage cell viability of A2780 cells at different time intervals upon treatment with hAMSC-CM. The percentage cell viability was significantly reduced after 48 h and 72 h of treatment with hAMSC-CM (*p* < 0.05 and *p* < 0.01, respectively). (**b**) MMP assay showing significantly enhanced (*p* < 0.01) JC-1 (monomer/aggregate) ratio after 72 h of treatment with hAMSC-CM as compared to non-treated controls. (**c**) Reactive oxygen species (ROS) formation increased (*p* < 0.05) after 72 h of treatment with hAMSC-CM as compared to controls. (**d**) Cell-cycle analysis showing that hAMSC-CM-treated cells exhibited increased S-phase population and decreased G0/G1-phase population from 48 h onward, while the G2/M-phase population also increased slightly after 72 h. (**e**) Representative data of terminal deoxynucleotidyl transferase dUTP nick-end-labelling assay showing increased apoptosis rates in A2780 cells after 72 h of treatment with hAMSC-CM. (**f**) Expression of intrinsic apoptosis-signaling molecules, upregulation of pro-apoptotic proteins (BAX, cytosolic cytochrome-c, CAS9, and CAS3), and downregulation of anti-apoptotic BCL2, indicating activation of the intrinsic apoptosis-signaling pathway. The experiments were repeated at least three times. *p < 0.05, **p < 0.01 and ***p < 0.001.

Treatment with hAMSC-derived CM altered cell proliferation through enhanced oxidative stress and reduced mitochondrial membrane potential (MMP). During the course of determining optimal treatment parameters, we observed that supplementation with hAMSC-derived CM did not exhibit changes in pH of culture medium; however, as shown in Supplementary Fig. S1, cell-viability assays revealed that viability began to decrease following treatment with 20% CM, with optimum inhibition observed at 25% CM supplementation. As shown in Fig. 1a, significant declines in cell viability were observed at 48 h (20% reduction) and 72 h (40% reduction) after treatment with 25% CM (*p* < 0.05 and *p* < 0.01, respectively) as compared with the control.

To evaluate the effects of cell apoptosis according to hAMSC-derived CM treatment, we examined the generation of reactive oxygen species (ROS) and MMP in CM-treated A2780 cells. As shown in Fig. 1b, 25% CM treatment resulted in an enhanced JC-1 monomer/aggregate ratio of 65% relative to control cells, as well as significant increases of ROS to 30% more than observed in control cells (*p* < 0.05) (Fig. 1c). Cell cycle analysis at 48 h following CM treatment revealed significant (*p* < 0.05) increases in the number of S-phase cells and decreases in G0/G1- and G2/M-phase cells (Fig. 1d). However, 72 h of CM treatment resulted in increases in both S-phase and G2/M-phase cells, and significant (*p* < 0.05) decreases in G0/G1-phase cells. These results indicated that hAMSC-CM restrained proliferation and blocked cell cycle of A2780 cancer cells.

### hAMSC-CM treatment activates the intrinsic apoptosis pathway in A2780 cells

To determine the induction of cell death following hAMSC-derived CM treatment, first we performed tetramethylrhodamine (TMR)-red assay and measured the percentage of apoptotic cells. Fluorescence-activated cell sorting analysis revealed significant (*p* < 0.05) increases in the percentage of apoptotic cells following CM treatment as compared with control cells (Fig. 1e). Second, we decided to detect the intrinsic apoptosis pathway-related anti- and pro-apoptotic molecules expression in CM-treated A2780 cells. In CM-treated A2780 cells, expression of anti-apoptotic protein BCL2 was downregulated as compared with control cells, while, several pro-apoptotic molecules, including phosphorylated p53, BAX, CASP9, CASP3, and cytosolic Cytochrome-c expression were significantly upregulated or activated. Additionally, the BAX/BCL2 ratio and active-/pro-CASP3 ratios increased by several folds in CM-treated cells relative to controls (Fig. 1f). Supplementary Fig. S2 shows full-length of gel images. These findings indicated that hAMSC-CM treatment initiated activation of the intrinsic apoptosis pathway in A2780 cells.

### Identification and characterization of hAMSC-CM-derived exosomes, and incorporation of exosomes within A2780 cells

**Figure 2:**
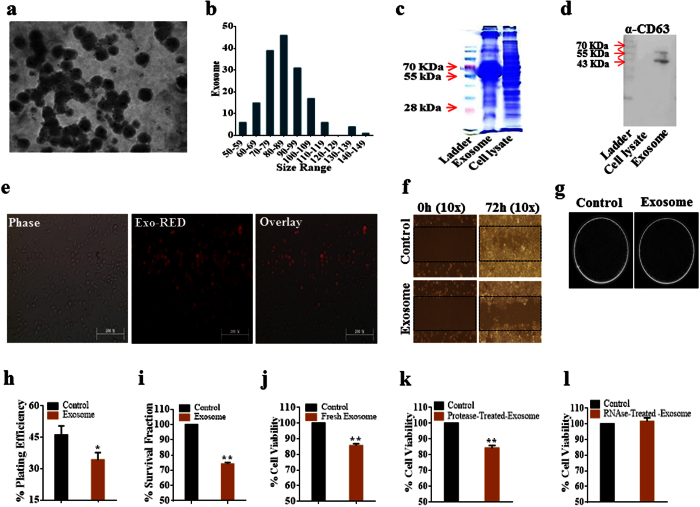
hAMSC-CM-derived exosomes mediated inhibition of A2780 cancer cells. (**a**) TEM results showing spherical exosomes isolated from hAMSC-CM. (**b**) Bar diagram representing the size distribution of exosomes ranging between 50 nm and 150 nm in diameter, with the majority between 70 nm and 100 nm in diameter. (**c**) Coommassie Blue staining of SDS-PAGE results indicating protein distributions in exosome and cell lysates. (**d**) Expression of exosome-specific marker (α-CD63) in exosome-derived proteins detected by western blot. (**e**) Internalization of glow-exosomes (fluorescence-tagged exosomes) by A2780 cells. (**f**) Representative photo of wound-healing assay. Exosome-treated cells exhibited decreased wound-healing capacity as compared with non-treated control cells. (**g**) Colony formation ability of exosome-treated cells declined as compared to non-treated control cells. (**h**) The percentage plating efficiency decreased significantly (*p* < 0.05) following exosome treatment as compared with control cells. (**i**) The percentage survival fraction decreased significantly (*p* < 0.01) in cells treated with hAMSC-CM-derived exosomes as compared with non-treated controls. (**j**) Cell viability of A2780 cells reduced significantly (*p* < 0.01) following exosome treatment. (**k**) The percentage cell viability continued to decrease (*p* < 0.01) following treatment with protease-digested exosome lysate as compared with controls. (**l**) The percentage cell viability in cells treated with RNase-digested exosome lysate did not decline, but rather resulted in slight increases in viability. The experiments were repeated at least three times. *p < 0.05 and **p < 0.01.

Then, we investigated whether hAMSC-CM-derived exosomes were responsible for the induction of apoptosis observed on CM-treated A2780 cells. Figure 2a shows hAMSC-CM-derived exosomes detected by transmission electron microscopy (TEM). Exosomes appeared as spheres having a size distribution from 50 nm to 150 nm, although the majority appeared to be between 70 nm and 100 nm (Fig. 2b). Next, we extracted the total proteins from exosomes and visualized the results with Coommassie Blue Staining (Fig. 2c). As shown in Fig. 2d, CD63, which is an exosome protein marker, was detectable in the exosomes-derived lysate, verifying the status of these structures as exosomes. Finally, we inspected whether exosome can internalize into A2780 cancer cells. To visualize, exosomes were labeled using Exo-red stain kit, which signifies binding with exosomal RNAs. As expected, CM-derived exosomes shows extensive internalization into A2780 cancer cells (Fig. 2e), indicating that hAMSC-CM-derived exosomes were successfully absorbed by the cells and might participate in deciding the fate of A2780 cancer cells.

### hAMSC-CM-derived exosomes reduced cell viability, wound-repair capacity, and colony formation ability of A2780 & SKOV-3 ovarian cancer cells

To determine the influence of hAMSC-CM-derived exosomes internalization on ovarian cancer cells, we treated A2780, SKOV-3 and CAOV-3 ovarian cancer cells using hAMSC-CM-derived exosomes and found that exosomes treatment had inhibitory influence on A2780 and SKOV-3 cells, while there was no remarkable change in CAOV-3 cells. We examined the wound-repair capacity (Fig. 2f) and colony formation ability (Fig. 2g) of A2780 cells. As shown in Fig. 2h,i and j, we observed significant reductions in cell-plating efficiency, cell-survival fraction, and cell viability following exosome internalization as compared to control cells (*p* < 0.05, *p* < 0.01 and *p* < 0.01, respectively). To determine the effects of A2780 cell incubation with either CM, exosomes, or both, we observed that incubation with exosomes alone caused a 28% loss in cell viability as compared to control cells, whereas incubation with exosomes and CM resulted in a 33% decrease in cell viability (*p* < 0.01; Supplementary Fig. S3). Similarly, cells treated with CM alone decreased the wound-healing capacity of the cells relative to control cells, while cells that internalized exosomes or both exosomes and CM, exhibited far less efficiency in wound-healing (Supplementary Fig. S3). Control cells exhibited ~46% plating efficiency, whereas cells that treated with CM or exosomes or else both exosomes and CM, exhibited 30% to 35% plating efficiency (*p* < 0.05, *p* < 0.05, and *p* < 0.01, respectively; Supplementary Fig. S4). Similarly, cell survival are also decreased by 30% to 35% in cells incubated with CM or those that had internalized CM-derived exosomes (*p* < 0.05 and *p* < 0.01, respectively) relative to levels observed in control cells (Supplementary Fig. S4). As presented in Supplementary Fig. S5, treatment using hAMSC-CM-derived fresh exosomes restrained proliferation of SKOV-3 cells by around 20%; similarly, protease-digested exosomes treatment also caused approximately 20% viability declination of SKOV-3 cells, compared to the control cells. In contrast, RNase-digested exosomes could not display inhibitory influence on SKOV-3 cells. These results indicated that hAMSC-CM-derived exosomes might be potential inhibitors against proliferation of A2780 and SKOV-3 ovarian cancer cells.

### A2780 cells responded dose- and time-dependently upon hAMSC-CM-derived exosomes treatment

As shown in Supplementary Fig. S6, dose-dependent treatment using hAMSC-CM-derived exosomes and viability of A2780 ovarian cancer cells demonstrated a negative correlation, higher the concentration of exosomes, lower the cell viability. More clearly, gradual inhibitions of cell proliferation recorded with gradual increase of doses of hAMSC-CM-derived exosomes. Likewise, viability of A2780 cells significantly (p < 0.01) reduced at 24 h incubation with one U/mL hAMSC-CM-derived exosomes, and the maximum inhibition was recorded at 48 h of treatment.

### hAMSC-CM-derived exosomal miRNAs inhibit A2780 cells proliferation

We then investigated whether exosomal protein or RNA was responsible for the anti-proliferative effects observed on A2780 cells. As shown in Fig. 2k, incubation of A2780 cells with protease-digested exosome lysates significantly reduced cell viability (*p* < 0.01) to a degree similar as that observed upon internalization of intact CM-derived exosomes. However, similar incubation with lysates from RNase-digested exosomes did not inhibit A2780 cell proliferation (Fig. 2l), but rather caused slight increases in cell viability. These findings suggested that exosomal RNA was responsible for inhibiting A2780 cell viability.

### Next-generation sequencing (NGS) confirmed oncogene-related miRNAs in hAMSC-CM-derived exosomes

**Figure 3:**
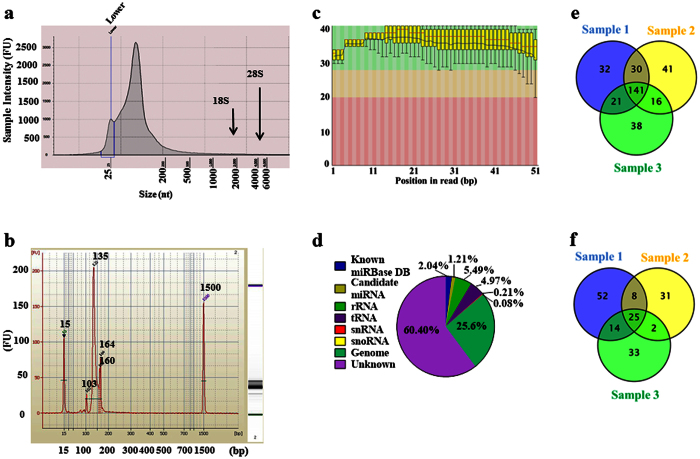
Small-RNA library preparation and NGS of exosomal RNA derived from hAMSC-CM. (**a**) Analysis of freshly isolated exosomal RNA indicated no peaks detected at 18 S or 28 S positions; however, exosomal RNA samples were enriched with small RNA. (**b**) Analysis showing preparation of the small-RNA library from exosomal RNA. (**c**) Sequence-quality data for exosomal RNA. (**d**) Pie chart illustrating the overall composition of exosomal RNA, with ~2% known miRNAs and 1.2% potential candidate miRNAs. (**e**) Venn diagram showing the distribution of known miRNA detected in three different replication samples and the 141 miRNAs common among all three samples. (**f**) Venn diagram representing the distribution of potential candidate miRNA in different samples and the 25 miRNAs common among all three samples. The experiments were repeated at least three times.

Presence of small RNA molecules in exosomes was confirmed by verifying the integrity of exosomal RNA (Fig. 3a), as well as by successful preparation of small RNA library (Fig. 3b) from hAMSC-CM-derived exosomal miRNAs. To assess whether miRNAs were present as precursors or fully processed transcripts, we examined length distributions of the miRNA reads and found that the majority of hAMSC-derived exosomal miRNA sequences ranged between 18 and 25 nucleotides in length (Supplementary Table S1).

Aiming to receive a quick impression about the quality of raw sequence data delivered by high throughput sequencing pipelines, quality control check-up was performed by FastQC (http://www.bioinformatics.babraham.ac.uk/projects/fastqc/) with a quality filter Q-score > 50 (Fig. 3c). Total reads were detected between 20,633,027 and 22,988,601, with a clustered read count between 687,897 and 765,338 in all three samples where the detected mapped and unmapped sequences were ~2% and ~98%, respectively. We determined that a substantial proportion of the cellular RNA content from hAMSC-derived exosomes was small-RNA, with ~2% constituting known miRNA and 1.2% potential candidate miRNA. The data also indicated the presence of ribosomal RNA, transfer RNA, small nuclear RNA, and small nucleolar RNA (Fig. 3d).

We found 141 known miRNAs (Fig. 3e) and 25 novel potential miRNAs (Fig. 3f), which were common in all three replicates. The most abundantly expressed known miRNA was hsa-miR-4792, which had read counts of 361010, 381745, and 381918 in the three replicates, respectively (Table 1). However, role of hsa-miR-4792 in cancer have not reported in literature and, most probably, remains undefined. The second and third most abundant miRNAs were hsa-miR-320b and hsa-miR-320a, which belong to the hsa-miR-320 family known for its roles in different molecular-and oncogene-signalling pathways^8^. Various members of the hsa-miR-127, hsa-miR-486, hsa-miR-423, hsa-miR-181, hsa-miR-423, hsa-miR-1246, hsa-miR-26, and hsa-miR-378 families were also observed among the 20 most abundant miRNAs, with each widely investigated and reported as having roles in cancer-regulatory activity^9^. Several other miRNAs which belong to top 20 known exosomal miRNAs, have not been extensively investigated for their oncogene regulatory properties; these miRNAs include hsa-miR-7704, hsa-miR-6087, hsa-miR-22-3p, hsa-miR-4466, hsa-miR-4532, hsa-miR-7641, hsa-miR-4448, hsa-miR-3960, and hsa-miR-3687 (Table 1).

The 141 known miRNAs belong to 104 different miRNA families, with 20 families containing two or more miRNAs found in all three replicates. Among them, eight miRNAs (Supplementary Table S1) belong to the hsa-let-7 family, which are recognized suppressor miRNAs in many types of cancer^10^. As demonstrated in Supplementary Table S1, five miRNAs belong to the hsa-miR-1273 family, which does not have well-documented anti-/pro-cancer properties, while among detected four members of the hsa-miR-30 family, hsa-miR-30c is reportedly involved promoting the invasiveness of metastatic breast cancer cells^11^. Five families (hsa-miR-320, hsa-miR-181, hsa-miR-29, hsa-miR-125, and hsa-miR-151) were each associated with three miRNAs found here. While members of the hsa-miR-320^8^ and hsa-miR-125 families^12^ are reported as having anticancer properties, miR-151 family members are documented as exhibiting pro-cancer^13^ functions. Member(s) of the hsa-miR-181^14^ and hsa-miR-29 families^15^ exhibit both anticancer and pro-cancer regulation under different clinical conditions, while members of hsa-miR-126^16^, hsa-miR-129^17^, hsa-miR-136^18^, hsa-miR-204^19^, hsa-miR-663^20^, hsa-miR-99^21^, hsa-miR-378^22^, hsa-miR-92^23^, and hsa-miR-409^24^ families are recognized as having anticancer properties under different clinical conditions. Families that contained at least one miRNA found in hAMSC-CM-derived exosomes reportedly exhibit antagonistic roles in numerous cancer types and include hsa-miR-486^25^, hsa-miR-100^26^, hsa-miR-101^27^, hsa-miR-124^28^, hsa-miR-1285^29^, hsa-miR-134^30^, hsa-miR-138^31^, hsa-miR-139^32^, hsa-miR-143^33^, hsa-miR-186^34^, hsa-miR-193^35^, hsa-miR-205^36^, hsa-miR-222^37^, hsa-miR-338^38^, hsa-miR-339^39^, hsa-miR-424^40^, hsa-miR-127^41^, hsa-miR-26^42^, hsa-miR-103^43^, hsa-miR-107^44^, hsa-miR-128^45^, hsa-miR-140^46^, hsa-miR-144^47^, hsa-miR-153^48^, hsa-miR-192^49^, hsa-miR-212^50^, hsa-miR-218^51^, hsa-miR-23^52^, hsa-miR-25^53^, hsa-miR-370^54^, hsa-miR-375^55^, hsa-miR-381^56^, hsa-miR-410^57^, hsa-miR-41^58^, and hsa-miR-93^59^. These NGS data revealed an enriched miRNA population in hAMSC-CM-derived exosomes that are likely involved in the regulation of different types of cancer.

### Functional enrichment analysis of exosomal miRNAs

**Figure 4:**
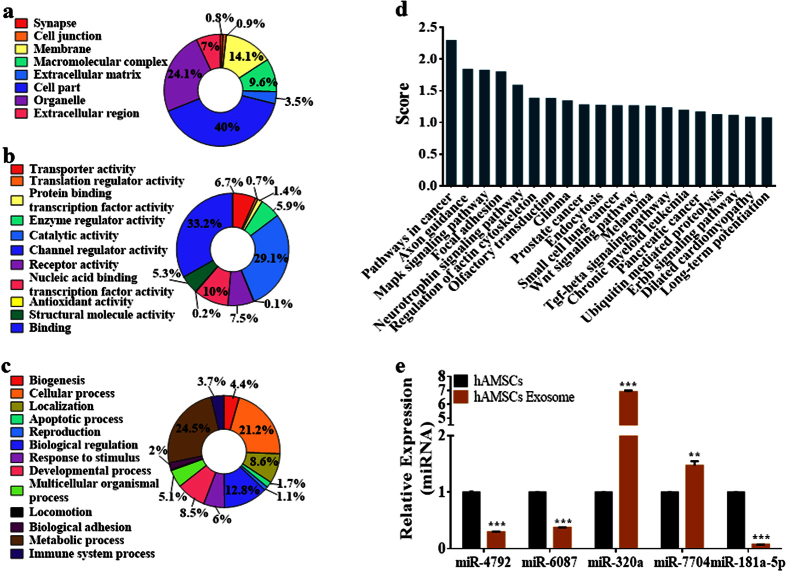
*In silico* GO and KEGG-pathway enrichment analysis of miRNAs detected in hAMSC-CM-derived exosomes. (**a**) Go analysis indicated that ~40% of the targeted genes were associated with cellular components, including 24% with organelles. (**b**) GO molecular functions analysis resulted in 33% involved as binding molecules and 29% with catalytic activity. (**c**) GO terms associated with biological processes were more diversified, with 25% of the target molecules involved in metabolic processes, 21% in cellular processes, 13% in biological regulation, 9% in localization, 8% in developmental processes. (**d**) KEGG-pathway enrichment indicated ‘Pathways in cancer’ as the top-scoring pathway among 173 KEGG pathways. (**e**) qRT-PCR validation of miRNA expression in exosomes, and host cells (hAMSCs). The miRNA-validation experiments were repeated at least three times. *p < 0.05, **p < 0.01 and ***p < 0.001.

To broaden our search for potential miRNAs, gene ontology (GO) functional enrichment analysis was performed on 8195 genes predicted as targets of the 141 known miRNAs found in CM-derived exosomes (Supplementary Spreadsheet S1). GO analysis indicated that approximately 40% of the targeted genes were associated with cellular components, including 24% with organelles, 14% with the cell membrane, 10% with macromolecular complexes, 7% with extracellular regions, and 3% with the extracellular matrix. A small portion of molecules was also associated with cell junctions and synapses (Fig. 4a). Of the target genes associated with molecular functions, approximately 33% are involved as binding molecules, 29% with catalytic activity, and 5% to 10% of the remaining genes with enzyme regulation, nucleic acid binding/transcription-factor activity, receptor activity, structural activity, and transporter activity (Fig. 4b). GO terms associated with biological processes were more diversified, with 25% of the target molecules involved in metabolic processes, 21% in cellular processes, 13% in biological regulation, 9% in localization, 8% in developmental processes, 6% in stimuli response, and 5% in multicellular organismal processes. Other targeted molecules were reportedly associated with biogenesis, apoptosis, reproduction, growth, biological adhesion, immune response, cell killing, and locomotion (Fig. 4c).

### *In silico* pathway analysis revealed cancer-related targets associated with hAMSC-CM-derived exosomal miRNAs

According to Kyoto Encyclopaedia of Genes and Genomes (KEGG) pathway analysis, 173 pathways were detected as targets for the 141 miRNAs detected in CM-derived exosomes (Supplementary Table S2). Each of the pathways was targeted by multiple miRNAs, with five miRNAs targeting the lowest scoring pathway and 88 miRNAs targeting the top-scoring pathway, which was ‘Pathways in cancer’ (Fig. 4d). Molecules associated with many other signalling pathways were predicted as targets for exosomal miRNAs, including the cancer-survival pathways related to TP53, MAPK, WNT, JAK-STAT, PI3K-AKT, cAMP, mTOR, TGF-β, VEGF, and PPAR.

The target molecules predicted by BioCarta (http://www.genecarta.com/), including those related to cell death and BCL2-associated death promoter (BAD)-signalling, are presented in Supplementary Fig. S7. Twenty two molecules associated with ‘Death-signalling pathway’ were predicted as targets for the 141 miRNAs found in CM-derived exosomes and included BCL2, XIAP/IAP, CASP3, GAS2, APAF1, CASP7, CASP10, CYCS, CASP8, SPTAN1, LMNA, BIRC2, MAP3K14, FADD, NFKB1, TNFRSF10B, BID, CASP6, NFKBIA, RELA, and DFFB. Additionally, 20 molecules related to the BAD-signalling pathway included IGF1R, BCL2, IGF1, ADCY1, PIK3R1, MAPK1, KITLG, BCL2L1, YWHAH, PRKACB, RPS6KA1, KIT, PIK3CA, PRKAR2B, PRKAR2A, PIK3CG, AKT1, BAD, IL3, and PRKAR1A. These findings indicate that miRNAs found in CM-derived exosomes target molecules involved in both cancer-survival signalling and cell death pathways, suggesting potentially important roles in the regulation of cancer proliferation, progression, and metastasis.

### Validation of miRNA expression in hAMSC-CM-derived exosomes and host cells

The expression of five selected miRNAs (hsa-miR-4792, hsa-miR-6087, hsa-miR-320a, hsa-miR-7704, and hsa-miR-181a-5p) was confirmed in both of hAMSC-CM-derived exosomes and a host cell line by using quantitative reverse-transcription polymerase chain reaction (qRT-PCR). All five miRNAs were detected in both exosomes and the host cell line, with three miRNAs (hsa-miR-4792, hsa-miR-6087, and hsa-miR-181a-5p) downregulated and two miRNAs (hsa-miR-320a and hsa-miR-7704) upregulated in exosomes as compared with the host cell line (Fig. 4e). These data indicated that exosomal miRNA expression could reflect the miRNA expression of host cell line in terms of miRNA population diversity, but may not reflect the level of expression of different miRNAs.

### Validation of target molecules associated with cancer-specific pathways

**Figure 5:**
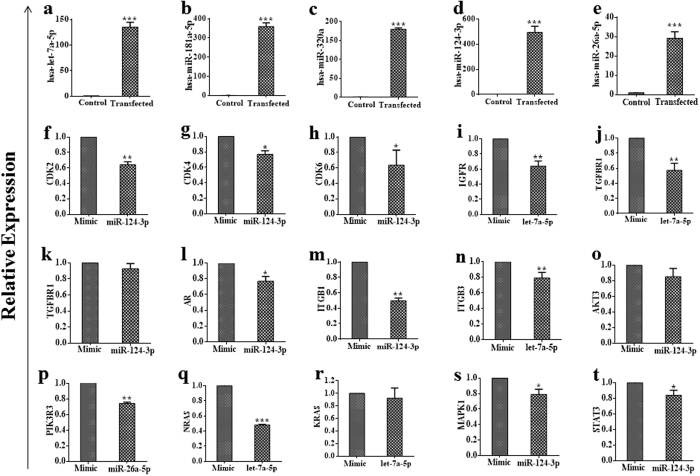
Validation of targets for different miRNAs in KEGG ‘Pathways in cancer’. (**a**–**e**) Confirmation of specific miRNA overexpression in transfected cells as compared with control cells. (**f–h**) Members of the CDK family found to be downregulated in has-miR-124-3p transfected cells. (**i**–**n**) Different cytokines and cytokine receptors targeted by different miRNAs. (**o**–**t**), Survival signaling-pathway molecules found to be downregulated following transfection of different miRNA as compared to negative-control cells. The experiments were repeated at least three times. *p < 0.05, **p < 0.01 and ***p < 0.001.

For target validation, each of the miRNAs (hsa-let-7a-5p, hsa-miR-181a-5p, hsa-miR-320a, hsa-miR-124-3p, and hsa-miR-26a-5p) was transfected into separate A2780 cells, respectively. Overexpression of specific miRNAs in each transfected cells were confirmed using qRT-PCR, with cells transfected with ‘*mir* Vana™ miRNA Mimic negative Control’ considered as an experimental control. The mimic-miRNA transfected cells exhibited up to 500-folds greater levels of expression relative to levels observed in the negative-control cells (Fig. 5a–e). We then assessed changes in the expression of selected target genes in miRNAs-transfected cells versus those in negative-control cells, to verify miRNA targets. Our results revealed that several members of the cyclin-dependent kinase (CDK) family, including CDK2, CDK4, and CDK6 (Fig. 5f–h), were targeted by hsa-miR-124-3p. Furthermore, several cytokines and cytokine receptors genes were also targeted by different exosomal miRNAs; these genes include IGFR, TGFBR1, AR, ITGB1, and ITGB3, which were downregulated by hsa-let-7a-5p/hsa-miR-124-3p (Fig. 5i–n). Several other molecules involved in cancer-survival signalling, including PIK3R, RAS, MAPK, and STAT, were also significantly dysregulated upon miRNA transfection in A2780 cells (Fig. 5o–t), respectively. The validated target molecules agreed with the predicted targets associated with KEGG-specific cancer-related pathways for the miRNAs identified from CM-derived exosomes (Supplementary Fig. S8). These results suggested that overexpression of specific exosomal miRNAs dysregulated molecules related to cell-cycle progression, cytokine and cytokine-receptor expression, and cancer-survival pathways.

### Internalization of hAMSC-CM-derived exosomes upregulates pro-apoptotic molecules in A2780 cells

**Figure 6:**
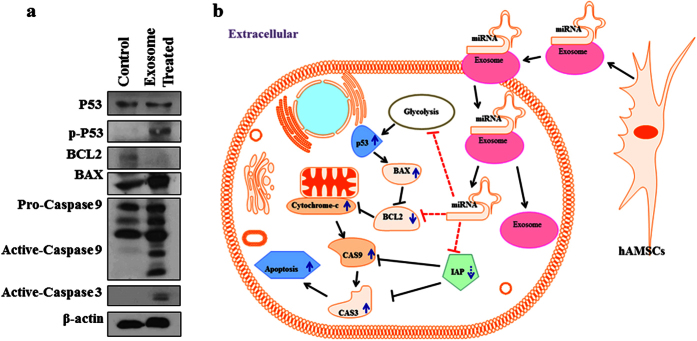
Potential mechanism of apoptosis activation in exosome-treated A2780 cancer cells. (**a**) Exosome-treated cells displayed upregulated expression of pro-apoptotic molecules and downregulated expression of anti-apoptotic BCL2. (**b**) hAMSC-CM-derived exosome inhibition of A2780 cell proliferation. hAMSCs release exosomes in CM, where exosomes function as carriers of MSC-secreted miRNAs. Upon internalization by cancer cells, miRNA is released by exosomes to target genes expressing different molecules associated with cancer proliferation, thereby disrupting cancer metastasis. The experiments were repeated at least three times.

Immunoblotting data in Fig. 6a showed upregulated expression of several pro-apoptotic signalling molecules, including phosphorylated p53, BAX, activated CASP9, and activated CASP3 in A2780 cells that had internalized CM-derived exosomes as compared with control cells. The full-length gel images are provided in Supplementary Fig. S9. In contrast, anti-apoptotic BCL2 was downregulated in these cells relative to control cells. These data indicated that internalization of CM-derived exosome resulted in activation of the intrinsic apoptosis pathway in A2780 cells.

## Discussion

MSCs are important regulators of biological processes such as healing, regeneration, maintenance, and remodeling^60^, under both normal and disease conditions. MSCs also play important roles in cancer biology; however, their ability to suppress or promote cancer remains questionable^2^. The data presented here showed that exosomes derived from hAMSC-CM were important carriers of miRNAs involved in the regulation of certain cancer types. We showed that treatment of A2780 cells with hAMSC-CM reduced cell viability, increased ROS production, and reduced MPP, indicating that CM derived from hAMSCs, and specifically the components released by hAMSCs, could induce deterioration of mitochondrial integrity, promote oxidative stress, and suppress cell proliferation. Moreover, treatment with CM induced S-phase arrest of the cell cycle, which is an uncommon phenomenon. In most cases, chemically induced cell-cycle arrest occurs mainly in G0/G1- and/or G2/M-phases; however, activation of the mitochondria-mediated apoptosis pathway^61^ and downregulation of CDKs^62^ could induce S-phase arrest.

Our findings revealed that overexpression of hsa-miR-124-3p, an exosomal miRNA, led to the downregulation of different CDKs, such as CDK2, CDK4, and CDK6. Additionally, other CDK family members were also predicted as targets for other exosomal miRNAs. Therefore, exosomal miRNA-mediated dysregulation of CDKs could be an explanation for S-phase arrest of the cell cycle in A2780 cancer cells. Similarly, internalization of exosomes resulted in greater decreases in proliferation, wound-repair capacity, and colony formation ability in A2780 cells compared to treatment with hAMSC-CM alone, thereby establishing that hAMSC-CM-derived exosomes are important factors related to inhibition of A2780 cell proliferation. We also observed that while treatment of A2780 cells with protease-digested exosome lysate reduced cell viability, treatment with RNase-digested exosome lysate did not suppress cell proliferation, indicating that exosomal RNAs were the sources of inhibition. Additionally, detection of numerous known miRNAs^8^,^9^,^10^,^11^,^12^,^13^,^14^,^15^,^16^,^17^,^18^,^19^,^20^,^21^,^22^,^23^,^24^,^25^,^26^,^27^,^28^,^29^,^30^,^31^,^32^,^33^,^34^,^35^,^36^,^37^,^38^,^39^,^40^,^41^,^42^,^43^,^44^,^45^,^46^,^47^,^48^,^49^,^50^,^51^,^52^,^53^,^54^,^55^,^56^,^57^,^58^,^59^ by NGS verified their reported involvement in regulation of growth, progression, and metastasis in different cancers. Furthermore, we validated that selected miRNA target molecules were involved in regulating cell-cycle progression, cytokine and cytokine-receptor expression, and cancer survival, suggesting that hAMSC-CM-derived exosomes are carriers of cancer-regulatory miRNAs.

Exceptionally, hAMSC-CM derived exosomal miRNAs demonstrated outstanding diversity, which encompasses numerous anti-cancer miRNAs^8^,^9^,^10^,^11^,^12^,^13^,^14^,^15^,^16^,^17^,^18^,^19^,^20^,^21^,^22^,^23^,^24^,^25^,^26^,^27^,^28^,^29^,^30^,^31^,^32^,^33^,^34^,^35^,^36^,^37^,^38^,^39^,^40^,^41^,^42^,^43^,^44^,^45^,^46^,^47^,^48^,^49^,^50^,^51^,^52^,^53^,^54^,^55^,^56^,^57^,^58^,^59^, as well as new and poorly investigated miRNAs. In contrast, miRNAs, which are frequently detected in different cancers exosomes (include hsa-miR-105, hsa-miR-214, hsa-miR-92, hsa-miR-21, hsa-miR-29, hsa-miR-9, hsa-miR-222)^63^,^64^, were either absent or showed very nominal expression in hAMSC-CM derived exosomes. Interestingly, exosomes derived from metastatic-cancers might also display high expression for anti-cancer miRNAs such as hsa-let-7, hsa-miR-23, hsa-miR-224 and hsa-miR-921^64^, which is believed as an internal reformation technique of the cancer cells to retain malignant properties by sacking the promising threats outside. However, presence of such anti-cancer miRNAs could not resists the pro-cancer activity of metastatic-cancer-derived exosomes. Instead, metastatic-tumor-derived exosomes functions as tracking system for the future site of metastasis^65^. Specially, the cytokines receptors (such as integrin) carried by the tumor-derived exosomes might work as antenna to recognize the potential site of metastasis, for instance, exosomes possess integrin α6β4 and α6β1 caused metastasis of cancer to lung, while, integrin αvβ5 resulted metastasis to liver^65^. Therefore, miRNAs are not the only players behind exosomes, rather, components including cytokines and proteins, lipids and others carried by exosomes might perform substantial role to motivate exosomes for its terminal accomplishment.

The mechanism(s) by which exosomes derived by hAMSCs transport different molecules, including miRNAs, to surrounding cells/tissues is illustrated in Fig. 6b. Once internalized by cells/tissues, the newly introduced miRNAs could target different signalling molecules related to cell cycle progression, cytokine and oncogene expression, and cancer-related pathways, ultimately resulting cell cycle blocking, reduced viability, upregulation of pro-apoptotic molecules, and downregulation of anti-apoptotic molecules. Considering the limited influence on high-grade serous CAOV-3 ovarian cancer cells, which did not show any considerable change in proliferation and wound healing capacity upon treatment with hAMSC-CM-derived exosomes, it appears that their affects are not identical for all cancer types, also the influence might depend on the level of malignance or stage of cancer progression. For instance, Lin *et al*.^66^ showed that internalization of hAMSC-derived exosomes promoted migration and invasiveness of MCF-7 cancer cells. Therefore, our findings suggested that the regulatory role of hAMSC-CM-derived exosomes on cancer cells is highly variable depending upon the type of cancer and surrounding niche as well as the mutation and acquired-resistance status of the cancer cell lines.

In conclusion, internalized hAMSC-CM-derived exosomes inhibited the growth and proliferation of A2780 and SKOV-3 ovarian cancer cells, suggesting that the relationship between MSCs and cancer could be partially explained by the mechanisms involved with exosome activity, given that MSC-derived exosomes contain miRNAs, which could be involved in cancer suppression or progression. Exosome influence could be defined by the enriched miRNA cluster, cancer types, and the surrounding niche. While MSCs have the potential to act as both cancer suppressors and promoters, the terminal mode of action could be determined by MSC origin, cancer type, and cytokine expression. The phenotypic relationships observed between MSCs and various cancers might not be rigid, and, therefore, could be determined by the cumulative effects of molecules released by MSCs. Our data provide valuable insights into these relationships; however, further extensive investigation is required to investigate the roles of exosomal miRNAs in different cancer types.

## Materials and Methods

### Reagents

ExoQuick-TC exosome-isolation reagent (EXOTC50A-1), SeraMir exosome RNA-isolation kit (RA808A-1), exosome-specific marker anti-αCD63 antibody (EXOABCD63A-1), and florescence exosome-preparation reagent (EXDC300A-1) were from System Biosciences (SBI, Palo Alto, CA, USA). Penicillin-streptomycin (PS) solution, trypsin-EDTA solution, RPMI medium, and α-MEM media were obtained from Life Technologies GIBCO (Grand Island, NY, USA). Fetal bovine serum (FBS) was from Sigma-Aldrich (St. Louis, MO, USA). Antibodies used for immunoblotting were against phosphorylated p53 (Cell Signaling Technology, Beverly, MA, USA), BAX, BCL2, CASP9, CASP3, β-actin (Abcam, Cambridge, MA), and cytochrome c (ENZO Diagnostics Inc., Farmingdale, NY, USA).

### Conditional medium (CM) preparation

hAMSCs were kindly donated by Maria Biotech Co. Ltd. (Seoul, South Korea) and cultured in α-MEM media containing 10% FBS and 100 U/mL PS in a humidified incubator at 37 °C and 5% CO_2_. The hAMSC-CM was collected from cells passage between six and seven times. Briefly, 2 × 10^6^ cells were seeded per 10-cm culture dish and incubated overnight at 37 °C and 5% CO_2_ for proper attachment. Media was replaced with new media, and cells were cultured for an additional 48 h, after which the resulting medium was considered hAMSC-CM. The pH stability was confirmed using both an electric pH meter and pH paper. A total of 1 L CM was collected, filtered, mixed, and aliquoted in 15-mL and 50-mL tubes for storage at −20 °C.

### CM dosage determination and cell-viability assay

To detect the effect of hAMSC-CM on A2780 cell proliferation, a cell-viability assay was performed using 0% to 30% of CM supplementation to the fresh medium. A2780 cells were seeded (1 × 10^4^ cells/well) onto 96-well, flat-bottomed culture plates and incubated for 24 h at 37 °C in a 5% CO_2_ incubator. The medium was replaced with control (fresh) or treatment (0–30% CM) medium, and cell viability was detected using a cell counting kit-8 (CCK-8; Dojindo Laboratories, Tokyo, Japan). Absorbance was measured at 450 nm in a microtiter plate reader (Multiskan FC; Thermo Fisher Scientific, Waltham, MA, USA). Based on obtained results, 25% CM supplementation to fresh medium was used for all experiments.

### ROS detection

ROS formation was quantified using 2′,7′-dichlorodihydrofluorescein diacetate (H2-DCFDA; Sigma-Aldrich). Cells were incubated with 10 μM H2-DCFDA at 37 °C in a humidified CO_2_ incubator for 30 min. Cells were then washed and re-suspended in phosphate-buffered saline (PBS), followed by detection of fluorescence emission and excitation at 515 nm and 488 nm, respectively, using a Gemini EM (SpectraMAX; Molecular Devices, Sunnyvale, CA, USA).

### MMP assessment

MMP was detected by the cationic fluorescent indicator JC-1 (Molecular Probes, Eugene, OR, USA). Cells were incubated with 10 μM JC-1 at 37 °C for 15 min, washed with and re-suspended in PBS, and the fluorescence intensity was measured. MMP was expressed as the ratio of the fluorescence intensity of the JC1 monomers to JC1 aggregates. JC1 aggregates indicate red fluorescence for intact mitochondria, with emission at 583 nm, while JC1 monomers demonstrate green fluorescence in cytoplasm, with emission at 525 nm and excitation at 488 nm.

### Cell cycle

A2780 cells were seeded onto 6-well plates (30,000 cells/cm^2^) and allowed to grow overnight, after which the medium was replaced with control (fresh) or treatment (25% CM) medium every 24 h. Cells were harvested at 48 h and 72 h for cell cycle analysis, washed with PBS, fixed with 70% ethanol, and stored at −20 °C overnight. Fixed cells were washed again with PBS, treated with RNaseA solution, and stained with propidium iodide. Cell-cycle analysis was performed using BD FACSCalibur (BD Biosciences, San Jose, CA, USA). The raw data was analysed using ModFit software trial version (http://www.vsh.com/products/mflt/index.asp).

### TMR red assay

A2780 cells were seeded onto 6-well plates (30,000 cells/cm^2^) and allowed to grow overnight. Medium was replaced with control (fresh) or treatment (25% CM) medium, and the medium was replaced every 24 h. At 72 h, cells were harvested and apoptosis rate was detected using an *in situ* cell-death detection kit (TMR red; Roche Diagnostics, Indianapolis, IN, USA) according to manufacturer instructions.

### Immunoblotting

Radio-immunoprecipitation buffer was used to lyse the cells, and protein concentrations were measured with the BCA protein assay kit (Thermo Scientific). Proteins were resolved using 12% SDS-PAGE and transferred electrophoretically to polyvinylidene fluoride membranes. Membranes were incubated with 5% skim milk for 2 h at room temperature (RT), then overnight at 4 °C with primary antibodies, and 1 h with horseradish peroxidase-conjugated secondary antibody at RT. Immuno-reactivity was detected by enhanced chemiluminescence reagents.

### Exosome isolation and characterization

Exosomes were isolated according to manufacturer instructions (ExoQuick-TC kit). Briefly, ExoQuick-TC reagent was added to hAMSC-CM at a 1:5 ratio and mixed by inverting the tubes several times. The mixture incubated overnight at 4 °C, centrifuged at 1500 *g* for 30 min at RT, and exosomes were recovered. For TEM, an exosome-TEM grid was prepared according to manufacturer instructions (Exosome-TEM-easy kit; 101Bio, Palo Alto, CA, USA), and exosome particles were visualized using an Hitachi H7600 electron microscope (Hitachi, Tokyo, Japan) at 100 kV accelerating voltage.

### Cell-viability assay

Exosomes derived from each mL of CM were designated as 1 U exosomes. For viability assays, cells were treated with exosomes at one U/mL. When using protease- or RNase-digested exosomes, the control medium was supplemented with equal amounts of protease or RNase solution to avoid experimental error due to the presence of protease or RNase. Protease-digested exosomes were prepared by digesting 5 U exosomes with 100 μL trypsin (0.25%) at 37 °C for 30 min. RNase-digested exosomes were obtained by digesting 5 U exosomes with 100 μL RNaseA solution (100 μg/mL) at 37 °C for 30 min.

### Wound-repair assay

For detecting wound-repair capacity, cells were seeded (30,000 cells/cm^2^) on 12-well plates and incubated overnight at 37 °C at 5% CO_2_. Wounds were made using 10-μl pipette tips, cells were washed with PBS, and fed with either control (fresh) or different treatment combinations (25% CM, 1 U/mL exosomes, or 1 U/mL exosomes plus 25% CM).

### Colony formation assay

For colony formation assays, 1000 cells were seeded onto 60-cm cell-culture dishes and incubated overnight at 37 °C at 5% CO_2_. Medium was replaced with either control (fresh) or different treatment combinations (25% CM, 1 U/mL exosomes, or 1 U/mL exosomes plus 25% CM), and the cells were cultured for 2 weeks. Dishes containing colonies were stained with 0.05% (w/v) crystal-violet solution, including 1% methanol and 1% formalin, for 20 min at RT. Colony photos were taken using an ultraviolet table, and colonies were counted using OpenCFU software (http://opencfu.sourceforge.net/). Cell-plating efficiency and survival fraction was calculated using the following formula:


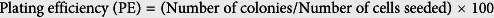



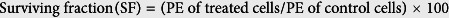


### Fluorescence-exosome preparation and internalization by cancer cells

Fluorescence exosomes were prepared and internalization of hAMSC-CM-derived exosomes by cancer cells was determined according to manufacturer protocol (EXDC300A-1, SBI, Palo Alto, CA, USA). Briefly, 50 μL of 10× Exo-Red was added to 500 μL of exosome suspension in PBS and mixed by inversion. The mixture was incubated at 37 °C for 10 min, followed by addition of 100 μL of ExoQuick-TC reagent to stop the reaction and incubation on ice for 30 min. The mixture was centrifuged for 3 min at 14,000 rpm in a microfuge, and the labelled-exosome pellet was re-suspended and delivered to cancer cells. Internalization was confirmed by fluorescence microscope.

### Exosomal RNA isolation

RNA was isolated from the exosome pellet using the SeraMir kit according to manufacturer protocol. Briefly, 350 μL of lysis buffer was added to 5 U exosome pellet, vortexed for 15 s, and incubated for 5 min at RT. The mixture was supplemented with 200 μL 100% ethanol, vortexed for 10 s, transferred to a spin column with collection tube, and centrifuged at 13,000 rpm for 1 min in a microfuge. The flow through was discarded, the spin column was washed twice with 400 μL wash buffer, and transferred to a new collection tube. Exosome-derived RNA was collected in 30 μL elution buffer and stored at −80 °C for downstream analysis.

### RNA analysis and small-RNA library preparation

RNA concentration was measured using a NanoDrop instrument (Thermo Scientific) and sent to Macrogen (Seoul, Korea) for small-RNA sequencing. Briefly, exosomal RNA integrity was detected by Agilent Technologies 2100 Bioanalyzer (Agilent Technologies, Santa Clara, CA, USA). Small-RNA libraries were prepared by adapter ligation, reverse transcription, PCR amplification, and pooled-gel purification. Small-RNA library size was confirmed by checking the template-size distribution using a DNA 1000chip and an Agilent 2100 Bioanalyzer (Agilent Technologies). To achieve the highest data quality, the prepared libraries were quantified using qPCR according to the Illumina qPCR Quantification Protocol Guide. We used the rapid library standard quantification solution and calculator (Roche) to generate a standard curve of fluorescence readings and calculate the library sample concentration.

### Sequence-quality check and data analysis

Sequence quality was verified by FastQC (http://www.bioinformatics.babraham.ac.uk/projects/fastqc) in order to determine data quality. Unique sequences found for known miRNAs after clustering were verified using the miRBase database (www.mirbase.org/) and BLAST (http://blast.ncbi.nlm.nih.gov/Blast.cgi). Trimmed reads were considered miRNA based on having 100% identical and full-length miRNA sequences as compared with those in the miRBase database. Unmatched trimmed reads not found in miRBase were compared against the ncRNA database Rfam (http://rfam.sanger.ac.uk/). Unmatched trimmed reads to non-miRNA in the Rfam database were considered potential novel miRNAs.

### miRNA detection and target prediction

Commonly expressed miRNAs among the three samples were sorted using Venny 2.0.2 BioinfoGP (http://bioinfogp.cnb.csic.es/tools/venny/). Targeted genes were predicted using the miRsystem RNA database (http://mirsystem.cgm.ntu.edu.tw/)^67^, which integrates seven miRNA-target gene-prediction programs, including DIANA, miRanda, miRBridge, PicTar, PITA, rna22, and TargetScan. Target genes predicted by at least three programs were considered for further analysis.

### GO and KEGG-pathway enrichment analysis

GO functional enrichment analysis was performed to verify predicted target genes against the PANTHER DNA database (http://pantherdb.org/). KEGG and BioCarta analyses were performed against the miRsystem RNA database, which integrates information from several pathway prediction programs, including KEGG (http://www.kegg.jp/)^68^, BioCarta (http://cgap.nci.nih.gov/Pathways/BioCarta_Pathways), Reactome (http://www.reactome.org/), and pathway interaction database (http://www.ndexbio.org/).

### Validation of miRNA expression in exosomes by qRT-PCR

Sequence validation to confirm miRNA in hAMSC-CM-derived exosomes was performed by qRT-PCR according to the instructions provided with the Mir-X miRNA qRT-PCR SYBR kit (Clontech Laboratories, Inc., CA, Mountain View, CA, USA). Briefly, cDNA was prepared from exosomal RNA by single-step polyadenylation and reverse-transcription reactions, followed by quantification of miRNA expression by qPCR. Known miRNA sequences were used as miRNA-specific 5′ primers, and mRQ 3′ primers supplied with the kit were used as 3′ primers. The U6 primer was detected as control. Primer sets are enlisted in Supplementary Table S3.

### Validation of miRNA targets

For validation of miRNA-target molecules in KEGG ‘Pathways in cancer’, five synthetic miRNAs were purchased based on three strategies, 1) firstly, the miRNA should have target(s) in ‘pathways in cancer’, 2) miRNA having higher ‘read count’ were prioritized 3) a representative was selected in case of families expressed multiple members. Then, selected miRNAs were separately transfected into A2780 cancer cells using lipofectamine transfection reagents. After 72 h, total RNA was isolated using a Qiagen RNeasy kit (Qiagen, Hilden, Germany). Relative mRNA expression of selected target genes was detected by qRT-PCR. Cells transfected with ‘*mir* Vana™ miRNA Mimic negative Control’ were considered experimental controls. Primers used for RT-PCR are enlisted in Supplementary Table S3.

### Statistical analysis

Experiments were performed in triplicate, and statistical analysis was performed using one-way analysis of variance. Significance of differences between control and treated cells was determined by Student *t* test. Significance was determined as *p* < 0.05, *p* < 0.01, and *p* < 0.001.

## Additional Information

**Accession codes:** High-throughput sequencing data from non-coding exosomal RNAs were deposited in the National Center for Biotechnology Information Gene Expression Omnibus database (series accession no. GSE81151).

**How to cite this article**: Reza, A. M. M. T. *et al*. Human adipose mesenchymal stem cell-derived exosomal-miRNAs are critical factors for inducing anti-proliferation signalling to A2780 and SKOV-3 ovarian cancer cells. *Sci. Rep.* **6**, 38498; doi: 10.1038/srep38498 (2016).

**Publisher’s note:** Springer Nature remains neutral with regard to jurisdictional claims in published maps and institutional affiliations.

## Supplementary Material

Supplementary Tables and Figures

Supplementary Spreadsheet S1

## Figures and Tables

**Table 1:** List (with details) of top 20 known miRNAs and three potential-new miRNA and seven potential-candidate miRNAs that were detected in hAMSCs-CM derived exosomes.

Known miRNA								
miRNA	Precursor	Sequence	Precursor Description	miRBase	ENTREZGENE	Sample 1	Sample 2	Sample 3
hsa-miR-4792	hsa-mir-4792	cggugagcgcucgcuggc	Homo sapiens miR-4792 stem-loop	miRBase:hsa-miR-4792	ENTREZGENE:100616448	361010	381745	381918
hsa-miR-320b	hsa-mir-320b-1,hsa-mir-320b-2	aaaagcuggguugagagggcaa	Homo sapiens miR-320b-1 stem-loop,Homo sapiens miR-320b-2 stem-loop	miRBase:hsa-miR-320b	ENTREZGENE: 100313823	2628	2094	2046
hsa-miR-320a	hsa-mir-320a	aaaagcuggguugagagggcga	Homo sapiens miR-320a stem-loop	miRBase:hsa-miR-320a	ENTREZGENE: 407037	1645	1311	1293
hsa-miR-7704	hsa-mir-7704	cggggucggcggcgacgug	Homo sapiens miR-7704 stem-loop	miRBase:hsa-miR-7704		1470	971	1145
hsa-miR-127-3p	hsa-mir-127	ucggauccgucugagcuuggcu	Homo sapiens miR-127 stem-loop	miRBase:hsa-miR-127-3p	ENTREZGENE: 406914	943	836	807
hsa-miR-6087	hsa-mir-6087	ugaggcgggggggcgagc	Homo sapiens miR-6087 stem-loop	miRBase:hsa-miR-6087		783	831	885
hsa-miR-22-3p	hsa-mir-22	aagcugccaguugaagaacugu	Homo sapiens miR-22 stem-loop	miRBase:hsa-miR-22-3p	ENTREZGENE:407004	772	638	671
hsa-miR-4466	hsa-mir-4466	gggugcgggccggcgggg	Homo sapiens miR-4466 stem-loop	miRBase:hsa-miR-4466	ENTREZGENE:100616154	459	541	618
hsa-miR-486-5p	hsa-mir-486-1,hsa-mir-486-2	uccuguacugagcugccccgag	Homo sapiens miR-486 stem-loop,Homo sapiens miR-486-2 stem-loop	miRBase:hsa-miR-486-5p	ENTREZGENE:619554	581	529	453
hsa-miR-4532	hsa-mir-4532	ccccggggagcccggcg	Homo sapiens miR-4532 stem-loop	miRBase:hsa-miR-4532	ENTREZGENE:100616353	449	449	625
hsa-miR-7641	hsa-mir-7641-1,hsa-mir-7641-2	uugaucucggaagcuaagc	Homo sapiens miR-7641-1 stem-loop,Homo sapiens miR-7641-2 stem-loop	miRBase:hsa-miR-7641		518	490	428
hsa-miR-423-5p	hsa-mir-423	ugaggggcagagagcgagacuuu	Homo sapiens miR-423 stem-loop	miRBase:hsa-miR-423-5p	ENTREZGENE:494335	328	273	273
hsa-miR-181a-5p	hsa-mir-181a-1,hsa-mir-181a-2	aacauucaacgcugucggugagu	Homo sapiens miR-181a-1 stem-loop,Homo sapiens miR-181a-2 stem-loop	miRBase:hsa-miR-181a-5p	ENTREZGENE:406954	226	224	130
hsa-miR-4448	hsa-mir-4448	ggcuccuuggucuaggggua	Homo sapiens miR-4448 stem-loop	miRBase:hsa-miR-4448	ENTREZGENE:100616127	232	144	137
hsa-miR-423-3p	hsa-mir-423	agcucggucugaggccccucagu	Homo sapiens miR-423 stem-loop	miRBase:hsa-miR-423-3p	ENTREZGENE:494335	195	143	155
hsa-miR-3960	hsa-mir-3960	ggcggcggcggaggcggggg	Homo sapiens miR-3960 stem-loop	miRBase:hsa-miR-3960	ENTREZGENE:100616250	134	105	148
hsa-miR-1246	hsa-mir-1246	aauggauuuuuggagcagg	Homo sapiens miR-1246 stem-loop	miRBase:hsa-miR-1246	ENTREZGENE:100302142	122	104	128
hsa-miR-26a-5p	hsa-mir-26a-1,hsa-mir-26a-2	uucaaguaauccaggauaggcu	Homo sapiens miR-26a-1 stem-loop,Homo sapiens miR-26a-2 stem-loop	miRBase:hsa-miR-26a-5p	ENTREZGENE:407015	146	110	74
hsa-miR-378a-3p	hsa-mir-378a	acuggacuuggagucagaaggc	Homo sapiens miR-378a stem-loop	miRBase:hsa-miR-378a-3p	ENTREZGENE:494327	93	113	96
hsa-miR-3687	hsa-mir-3687-1,hsa-mir-3687-2	cccggacaggcguucgugcgacgu	Homo sapiens miR-3687-1 stem-loop,Homo sapiens miR-3687-2 stem-loop	miRBase:hsa-miR-3687	ENTREZGENE:100500815	104	70	84
**Potential New-miRNAs**								
**Probitional miRNA_Id**	**Mature Sequence**	**Precursor Sequence**	**Mature Length (nt)**	**Chr No.**	**Start**	**End**	**Precursor Length(nt)**	
chr12:109181601-109181645	agaggaggagaagaaacu	uaacuuuuccccuacucuugccauagagaggaggagaagaaacu	18	chr12	109181601	109181645	45	
chr7:148638583-148638650	ccccacaaccgcgcuugacu	gguccgaguguuguggguuauuguuaaguugauuuaacauugucuccccccacaaccgcgcuugacu	20	chr7	148638583	148638650	68	
Chr16:73092094-73092157	ggcggcggcggcggcggc	ggcggcggcggcgcggggccggggaggagggggcggcccgggcucggcggcggcggcggcggc	18	Chr16	73092094	73092157	64	
**Expected Candidate-miRNAs**								
chr8:56821963-56822004	gugggauuaugacugaac	gugggauuaugacugaacauguccaagucagaaucccacuc	18	chr8	56821963	56822004	42	
chr6:28678365-28678429	ggggguguagcucaguggu	ggggguguagcucagugguagagcgcgugcuuagcaugcacgaggcccuggguucaauccccag	19	chr6	28678365	28678429	65	
chr6:28726148-28726212	ggggguguagcucaguggu	ggggguguagcucagugguagagcacaugcuuugcaugugugaggccccggguucgauccccgg	19	chr6	28726148	28726212	65	
chr20:36065457-36065499	gagaguuugauccuggcuc	guggggguggggguaggaggauggagaguuugauccuggcuc	19	chr20	36065457	36065499	43	
chr20:61801606-61801662	gcauccuggggcuggaga	gcauccuggggcuggagagguugaggcugccaccccuccagaccagagggaugugg	18	chr20	61801606	61801662	57	
chrY:10035763-10035818	agaggugaaauucuuggac	aagagggauggcugggggcauucguauugugccacuagaggugaaauucuuggac	19	chrY	10035763	10035868	106	
chr11:122928783-122928833	cuuccuuggaugucugagugac	caccagaauucaagguguuggcaacuaccuuccuuggaugucugagugac	22	chr11	122928783	122928833	51	

## References

[b1] Reza A. M. M. T. . Keratinocyte growth factor and thiazolidinediones and linolenic acid differentiate characterized mammary fat pad adipose stem cells isolated from prepubertal Korean black goat to epithelial and adipogenic lineage. In Vitro Cell Dev Biol-Anim. 50, 194–206 (2014). 24101555 10.1007/s11626-013-9690-5

[b2] Honga I. S., Lee H. Y. & Kang K. S. Mesenchymal stem cells and cancer: friends or enemies? Mutat Res. 768, 98–106 (2014). 24512984 10.1016/j.mrfmmm.2014.01.006

[b3] Nomoto-Kojima N. . Interaction between adipose tissue stromal cells and gastric cancer cells *in vitro*. Cell Tissue Res. 344, 287–298 (2011). 21384185 10.1007/s00441-011-1144-3

[b4] Qiao L. . Suppression of tumorigenesis by human mesenchymal stem cells in a hepatoma model. Cell Res. 18, 500–507 (2008). 18364678 10.1038/cr.2008.40

[b5] Zimmerlin L., Park T. S., Zambidis E. T., Donnenberg V. S. & Donnenberg A. D. Mesenchymal stem cell secretome and regenerative therapy after cancer. Biochimie. 95, 2235–2245 (2013). 23747841 10.1016/j.biochi.2013.05.010 PMC3825748

[b6] Saadeldin I. M., Oh H. J. & Lee B. C. Embryonic-maternal cross-talk via exosomes: potential implications. Stem Cells Cloning. 8, 103–107 (2015). 26185458 10.2147/SCCAA.S84991 PMC4500606

[b7] Lin S. & Gregory R. I. MicroRNA biogenesis pathways in cancer. Nat. Rev. Cancer. 15, 321–333 (2015). 25998712 10.1038/nrc3932 PMC4859809

[b8] Wan L. Y. . miR-320 enhances the sensitivity of human colon cancer cells to chemoradiotherapy *in vitro* by targeting FOXM1. Biochem Biophys Res Commun. 457, 125–132 (2015). 25446103 10.1016/j.bbrc.2014.11.039

[b9] Schwarzenbach H., Nishida N., Calin G. A. & Pantel K. Clinical relevance of circulating cell-free microRNAs in cancer. Nat. Rev. Clin. Oncol. 11, 145–156 (2014). 24492836 10.1038/nrclinonc.2014.5

[b10] Boyerinas B., Park S. M., Hau A., Murmann A. E. & Peter M. E. The role of let-7 in cell differentiation and cancer. Endocr Relat Cancer. 17, F19–F36 (2010). 19779035 10.1677/ERC-09-0184

[b11] Dobson J. R. . hsa-mir-30c promotes the invasive phenotype of metastatic breast cancer cells by targeting NOV/CCN3. Cancer Cell Int. 14, 73, 10.1186/s12935-014-0073-0 (2014). 25120384 PMC4129468

[b12] Ferracin M. . miR-125b targets erythropoietin and its receptor and their expression correlates with metastatic potential and ERBB2/HER2 expression. Mol Cancer. 12, 130, 10.1186/1476-4598-12-130 (2013). 24165569 PMC4176119

[b13] McNally M. E. . Concomitant dysregulation of microRNAs miR1513p and miR126 correlates with improved survival in resected cholangiocarcinoma. HPB. 15, 260 264 (2013). 23458262 10.1111/j.1477-2574.2012.00523.x PMC3608979

[b14] Liu J., Shi W., Wu C., Ju J. & Jiang J. miR-181b as a key regulator of the oncogenic process and its clinical implications in cancer (Review). Biomed Rep. 2, 7–11 (2014). 24649060 10.3892/br.2013.199 PMC3917097

[b15] Jiang H., Guang Z., Wu J. H. & Jiang C. P. Diverse roles of miR-29 in cancer (Review). Oncology Reports. 31, 1509–1516 (2014). 24573597 10.3892/or.2014.3036

[b16] Xiong Y., Kotian S., Zeiger M. A., Zhang L. & Kebebew E. miR-126-3p inhibits thyroid cancer cell growth and metastasis, and is associated with aggressive thyroid cancer. PLoS ONE. 10, e0130496, 10.1371/journal.pone.0130496 (2015). 26244545 PMC4526518

[b17] Karaayvaz M., Zhai H. & Ju J. miR-129 promotes apoptosis and enhances chemosensitivity to 5-fluorouracil in colorectal cancer. Cell Death Dis. 4, e659, 10.1038/cddis.2013.193 (2013). 23744359 PMC3702282

[b18] Zhao H. . Expression of miR-136 is associated with the primary cisplatin resistance of human epithelial ovarian cancer. Oncol Rep. 33, 591–598 (2015). 25482209 10.3892/or.2014.3640

[b19] Shi L. . MiR-204 inhibits human NSCLC metastasis through suppression of NUAK1. Br. J. Cancer. 111, 2316–2327 (2014). 25412236 10.1038/bjc.2014.580 PMC4264457

[b20] Zang W. . miR-663 attenuates tumor growth and invasiveness by targeting eEF1A2 in pancreatic cancer. Mol Cancer. 14, 37, 10.1186/s12943-015-0315-3 (2015). 25744894 PMC4332743

[b21] Wang L. . miR99a and 99b inhibit cervical cancer cell proliferation and invasion by targeting mTOR signaling pathway. Med Oncol. 31, 934, 10.1007/s12032-014-0934-3 (2014). 24668416

[b22] Li H. . Clinical and biological significance of miR-378a-3p and miR-378a-5p in colorectal cancer. Eur J Cancer. 50, 1207–1221 (2014). 24412052 10.1016/j.ejca.2013.12.010

[b23] Ohyagi-Hara C. . miR-92a inhibits peritoneal dissemination of ovarian cancer cells by inhibiting integrin α5 expression. Am J Pathol. 182, 1876–1889 (2013). 23499550 10.1016/j.ajpath.2013.01.039

[b24] Liu M. . Downregulation of microRNA-409-3p promotes aggressiveness and metastasis in colorectal cancer: an indication for personalized medicine. J Transl Med. 13, 195, 10.1186/s12967-015-0533-x (2015). 10.1186/s12967-015-0533-x PMC4472171 26084278

[b25] Chen H. . Expression and prognostic value of miR-486-5p in patients with gastric adenocarcinoma. PLoS ONE. 10, e0119384, 10.1371/journal.pone.0119384 (2015). 25793394 PMC4368750

[b26] Zhou M. K., Liu X. J., Zhao Z. G. & Cheng Y. M. MicroRNA-100 functions as a suppressor by inhibiting Lgr5 expression in colon cancer cells. Mol Med Rep. 11, 2947–2952 (2015). 25483280 10.3892/mmr.2014.3052

[b27] Zhao S. . Loss of MicroRNA-101 Promotes epithelial to mesenchymal transition in hepatocytes. J. Cell. Physiol. 230, 2706–2717 (2015). 25808945 10.1002/jcp.24995

[b28] Shi X. B. . Tumor suppressive miR-124 targets androgen receptor and inhibits proliferation of prostate cancer cells. Oncogene. 32, 4130–4138 (2013). 23069658 10.1038/onc.2012.425 PMC4111479

[b29] Liu J. . miR-1285-3p acts as a potential tumor suppressor miRNA via downregulating JUN expression in hepatocellular carcinoma. Tumour Biol. 36, 219–225 (2015). 25230788 10.1007/s13277-014-2622-5

[b30] Zha R. . Genome-wide screening identified that miR-134 acts as a metastasis suppressor by targeting integrin β1 in hepatocellular carcinoma. PLoS ONE. 9, e87665, 10.1371/journal.pone.0087665 (2014). 24498348 PMC3912066

[b31] Golubovskaya V. M., Sumbler B., Ho B., Yemma M. & Cance W. G. MiR-138 and MiR-135 directly target focal adhesion kinase, inhibit cell invasion, and increase sensitivity to chemotherapy in cancer cells. Anticancer Agents Med Chem. 14, 18–28 (2014). 23438844 10.2174/187152061401140108113435 PMC3883917

[b32] Liu X. . MicroRNA-139-3p indicates a poor prognosis of colon cancer. Int J Clin Exp Pathol. 7, 8046–8052 (2014). 25550849 PMC4270559

[b33] Wei J. . miR-143 inhibits cell proliferation by targeting autophagy-related 2B in non-small cell lung cancer H1299 cells. Mol Med Rep. 11, 571–576 (2015). 25322940 10.3892/mmr.2014.2675

[b34] Cui G. . MiR-186 targets ROCK1 to suppress the growth and metastasis of NSCLC cells. Tumor Biol. 35, 8933–8937 (2014). 10.1007/s13277-014-2168-6 24894676

[b35] Yu T. . MicroRNA-193a-3p and -5p suppress the metastasis of human non-small-cell lung cancer by downregulating the ERBB4/PIK3R3/mTOR/S6K2 signaling pathway. Oncogene. 34, 413–423 (2015). 24469061 10.1038/onc.2013.574

[b36] Elgamal O. A. . Tumor suppressive function of mir-205 in breast cancer is linked to HMGB3 regulation. PLoS ONE. 8, e76402, 10.1371/journal.pone.0076402 (2013). 24098490 PMC3788717

[b37] Liu X. . MicroRNA-222 regulates cell invasion by targeting matrix metalloproteinase 1 (MMP1) and manganese superoxide dismutase 2 (SOD2) in tongue squamous cell carcinoma cell lines. Cancer Genom Proteom. 6, 131–139 (2009). PMC2890246 19487542

[b38] Xue Q. . MicroRNA-338-3p inhibits colorectal carcinoma cell invasion and migration by targeting smoothened. Jpn J Clin Oncol. 44, 13–21 (2014). 24277750 10.1093/jjco/hyt181

[b39] Zhang C. . MicroRNA-339-5p inhibits colorectal tumorigenesis through regulation of the MDM2/p53 signaling. Oncotarget. 5, 9106–9117 (2014). 25193859 10.18632/oncotarget.2379 PMC4253422

[b40] Ruiz-Llorente L., Ardila-González S., Fanjul L. F., MartínezIglesias O. & Aranda A. MicroRNAs 424 and 503 are mediators of the anti-proliferative and anti-invasive action of the thyroid hormone receptor beta. Oncotarget. 5, 2918–2933 (2014). 24796297 10.18632/oncotarget.1577 PMC4102780

[b41] Chen J., Wang M., Guo M., Xie Y. & Cong Y. S. miR-127 regulates cell proliferation and senescence by targeting BCL6. PLoS ONE. 8, e80266, 10.1371/journal.pone.0080266 (2013). 24282530 PMC3840165

[b42] Dang X. . MicroRNA-26a regulates tumorigenic properties of EZH2 in human lung carcinoma cells. Cancer Genet. 205, 113–123 (2012). 22469510 10.1016/j.cancergen.2012.01.002

[b43] Liang J. . MicroRNA-103a inhibits gastric cancer cell proliferation, migration and invasion by targeting c-Myb. Cell Prolif. 48, 78–85 (2015). 25530421 10.1111/cpr.12159 PMC6496034

[b44] Ji Y. . Decreased expression of microRNA107 predicts poorer prognosis in glioma. Tumour Biol. 36, 4461 4466 (2015). 25596705 10.1007/s13277-015-3086-y

[b45] Li B., Chen H., Wu N., Zhang W. J. & Shang L. X. Deregulation of miR128 in ovarian cancer promotes cisplatin resistance. Int J Gynecol Cancer. 24, 1381–1388 (2014). 25248111 10.1097/IGC.0000000000000252

[b46] Takata A. . MiRNA-140 acts as a liver tumor suppressor by controlling NFκB activity via directly targeting Dnmt1 expression. Hepatology. 57, 162–170 (2013). 22898998 10.1002/hep.26011 PMC3521841

[b47] Lin L. . MicroRNA-144 suppresses tumorigenesis and tumor progression of astrocytoma by targeting EZH2. Hum Pathol. 46, 971–980 (2015). 25907866 10.1016/j.humpath.2015.01.023

[b48] Shan N., Shen L., Wang J., He D. & Duan C. MiR-153 inhibits migration and invasion of human non-small-cell lung cancer by targeting ADAM19. Biochem Biophys Res Commun. 456, 385–391 (2015). 25475731 10.1016/j.bbrc.2014.11.093

[b49] Jin Y., Lu J., Wen J., Shen Y. & Wen X. Regulation of growth of human bladder cancer by miR-192. Tumour Biol. 36, 3791–3797 (2015). 25566965 10.1007/s13277-014-3020-8

[b50] Luo X. J. . MicroRNA-212 inhibits osteosarcoma cells proliferation and invasion by down regulation of Sox4. Cell Physiol Biochem. 34, 2180–2188 (2014). 25562164 10.1159/000369661

[b51] Li J., Li X., Wang J., Wang Y. & Qiu H. MicroRNA-218 increases cellular sensitivity to rapamycin via targeting rictor in cervical cancer. APMIS. 123, 562–570 (2015). 25908215 10.1111/apm.12387

[b52] Ishihara T. . Expression of the tumor suppressive miRNA-23b/27b cluster is a good prognostic marker in clear cell renal cell carcinoma. J Urol. 192, 1822–1830 (2014). 25014580 10.1016/j.juro.2014.07.001

[b53] Zoni E. . miR-25 modulates invasiveness and dissemination of human prostate cancer cells via regulation of αv- and α6-integrin expression. Cancer Res. 75, 2326–2336 (2015). 25858144 10.1158/0008-5472.CAN-14-2155

[b54] Yungang W., Xiaoyu L., Pang T., Wenming L. & Pan X. miR-370 targeted FoxM1 functions as a tumor suppressor in laryngeal squamous cell carcinoma (LSCC). Biomed Pharmacother. 68, 149–154 (2014). 24055400 10.1016/j.biopha.2013.08.008

[b55] Ye X. M. . Epigenetic silencing of miR-375 induces trastuzumab resistance in HER2-positive breast cancer by targeting IGF1R. BMC Cancer. 26, 14:134, 10.1186/1471-2407-14-134 (2014). PMC3974046 24571711

[b56] Xu Y. . Changes in the expression of miR-381 and miR-495 are inversely associated with the expression of the MDR1 gene and development of multi-drug resistance. PLoS ONE. 8, e82062, 10.1371/journal.pone.0082062 (2013). 24303078 PMC3841137

[b57] Shen J. . MicroRNA-410 suppresses migration and invasion by targeting MDM2 in gastric cancer. PLoS ONE. 9, e104510, 10.1371/journal.pone.0104510 (2014). 25136862 PMC4138091

[b58] Yamamoto K. . MiR-379/411 cluster regulates IL-18 and contributes to drug resistance in malignant pleural mesothelioma. Oncol Rep. 32, 2365–2372 (2014). 25231602 10.3892/or.2014.3481

[b59] Tang Q. . MicroRNA-93 suppress colorectal cancer development via Wnt/β-catenin pathway downregulating. Tumour Biol. 36, 1701–1710 (2015). 25371073 10.1007/s13277-014-2771-6

[b60] Reza A. M. M. T., Lee S., Shiwani S. & Singh N. K. KGF and BMP-6 intervene in cellular reprogramming and in mesenchymal-epithelial transition (MET) of 3T3L1 mouse adipose cells. Cell Biol Int. 39, 400–410 (2015). 25492426 10.1002/cbin.10407

[b61] Tang L. . Potent activation of mitochondria-mediated apoptosis and arrest in S and M phases of cancer cells by a broccoli sprout extract. Mol Cancer Ther. 5, 935–944 (2006). 16648564 10.1158/1535-7163.MCT-05-0476

[b62] Zhu H. . Induction of S-phase arrest and p21 overexpression by a small molecule 2[[3-(2,3-dichlorophenoxy)propyl] amino]ethanol in correlation with activation of ERK. Oncogene. 23, 4984–4992 (2004). 15122344 10.1038/sj.onc.1207645

[b63] Zhang J. . Exosome and exosomal microRNA: trafficking, sorting, and function. Genomics Proteomics Bioinformatics 13, 17–24 (2015). 25724326 10.1016/j.gpb.2015.02.001 PMC4411500

[b64] Falcone G., Felsani A. & D’Agnano I. Signaling by exosomal microRNAs in cancer. J Exp Clin Cancer Res. 34, 32, 10.1186/s13046-015-0148-3 (2015). 25886763 PMC4391656

[b65] Hoshino A. . Tumour exosome integrins determine organotropic metastasis. Nature 527, 329–335 (2015). 26524530 10.1038/nature15756 PMC4788391

[b66] Lin R., Wang S. & Zhao R. C. Exosomes from human adipose-derived mesenchymal stem cells promote migration through Wnt signaling pathway in a breast cancer cell model. Mol Cell Biochem. 383, 13–20 (2013). 23812844 10.1007/s11010-013-1746-z

[b67] Lu T.-P. . miRSystem: an integrated system for characterizing enriched functions and pathways of microRNA targets. PLoS ONE. 7, e42390, 10.1371/journal.pone.0042390 (2012). 22870325 PMC3411648

[b68] Kanehisa M. & Goto S. KEGG: kyoto encyclopedia of genes and genomes. Nucleic Acids Res. 28, 27–30 (2000). 10592173 10.1093/nar/28.1.27 PMC102409

