# Supplementary material for: Human adipose mesenchymal stem cell-derived exosomal-miRNAs are critical factors for inducing anti-proliferation signalling to A2780 and SKOV-3 ovarian cancer cells
Source: Sci Rep. 2016 Dec 8;6:38498. doi: 10.1038/srep38498 (PMC5143979; doi:10.1038/srep38498)
Supplement: Supplementary Tables and Figures [file srep38498-s1.pdf]

Supplementary Data

Human adipose mesenchymal stem cell-derived exosomal-miRNAs are critical factors for inducing anti-proliferation signalling to A2780 and SKOV-3 ovarian cancer cells

Abu Musa Md Talimur Reza, Yun-Jung Choi, Hideyo Yasuda & Jin-Hoi Kim*

Department of Stem Cell and Regenerative Biology, Humanized Pig Research Centre (SRC), Konkuk University, Seoul 143-701, Republic of Korea

*** Corresponding author**: Department of Stem Cell and Regenerative Biology, Humanized Pig Research Center (SRC), Konkuk University, Seoul 143-701, Republic of Korea.

E-mail address: jhkim541@konkuk.ac.kr

Running title: Exosomal-miRNAs intervention in ovarian cancer cells

Supplementary Table S1: Summary (with details) of miRNA expression detected in exosomes derived from hAMSC-CM.

Mature miRNA	Family Name	Sequence	Read Counts		
			Sample 1	Sample 2	Sample 3
hsa-miR-4792	hsa-miR-4792	cggugagcgcucgcuggc	361010	381745	381918
hsa-miR-320a	hsa-miR-320	aaaagcuggguugagagggcga	1645	1311	1293
hsa-miR-320b		aaaagcuggguugagagggcaa	2628	2094	2046
hsa-miR-320c		aaaagcuggguugagagggu	19	16	26
hsa-miR-26a-5p	hsa-miR-26	uucaaguaauccaggauaggcu	146	110	74
hsa-miR-378a-3p	hsa-miR-378	acuggacuuggagucagaagg	93	113	96
hsa-miR-7704	hsa-miR-7704	cggggucggcggcgacgug	1470	971	1145
hsa-miR-181a-5p	hsa-miR-181	aacauucaacgcugucggugagu	226	224	130
hsa-miR-181b-5p		aacauucauugcugucggugggu	28	26	8
hsa-miR-181c-5p		aacauucaaccugucggugagu	5	1	1
hsa-miR-127-3p	hsa-miR-127	ucggauccgucugagcuuggcu	943	836	807
hsa-miR-4466	hsa-miR-4466	gggugcgggccggcgggg	459	541	618
hsa-miR-423-3p	hsa-miR-423	agcucggucugaggccccucagu	195	143	155
hsa-miR-423-5p		ugaggggcagagagcgagacuuu	328	273	273
hsa-miR-1246	hsa-miR-1246	aauggauuuuuggagcagg	122	104	128
hsa-miR-22-3p	hsa-miR-22	aagcugccaguugaagaacugu	772	638	671
hsa-miR-7641	hsa-miR-7641	uugaucucggaagcuaagc	518	490	428
hsa-miR-3960	hsa-miR-3960	ggcggcggcggaggcggggg	134	105	148
hsa-let-7a-5p	hsa-let-7	ugagguaguagguuguauaguu	33	33	9
hsa-let-7b-5p		ugagguaguagguugugugguu	5	2	4
hsa-let-7c-5p		ugagguaguagguuguaugguu	12	9	10
hsa-let-7d-5p		agagguaguagguugcauaguu	3	1	2
hsa-let-7e-5p		ugagguaggagguuguauaguu	4	1	2
hsa-let-7f-5p		ugagguaguagauuguauaguu	32	18	16
hsa-let-7g-5p		ugagguaguaguuuguacaguu	3	5	1
hsa-let-7i-5p		ugagguaguaguuugugcuguu	9	6	3
hsa-miR-6087	hsa-miR-6087	ugaggcgggggggcgagc	783	831	885
hsa-miR-1273a	hsa-miR-1273	gggcgacaaagcaagacucuuucuu	16	4	4
hsa-miR-1273d		gaacccaugagguugaggcugcagu	23	13	5
hsa-miR-1273e		uugcuugaacccaggaagugga	1	8	7
hsa-miR-1273f		ggagauggagguugcagug	17	39	31
hsa-miR-1273g-3p		accacugcacuccagccugag	52	83	87
hsa-miR-4448	hsa-miR-4448	ggcuccuuggucuaggggua	232	144	137
hsa-miR-486-5p	hsa-miR-486	uccuguacugagcugccccgag	581	529	453
hsa-miR-129-2-3p	hsa-miR-129	aagcccuuaccccaaaaagcau	2	2	2
hsa-miR-129-5p		cuuuuugcggucugggcuugc	6	2	6
hsa-miR-4532	hsa-miR-4532	ccccggggagcccggcg	449	449	625
hsa-miR-619-5p	hsa-miR-619	gcugggauuacaggcaugagcc	74	61	83
hsa-miR-136-3p	hsa-miR-136	caucaucgucucaaaugagucu	6	9	4
hsa-miR-136-5p		acuccauuuguuuugaugaugga	3	3	3
hsa-miR-151a-3p	hsa-miR-151	cuagacugaagcuccuugagg	12	14	17
hsa-miR-151a-5p		ucgaggagcucacagucuagu	3	4	3
hsa-miR-151b		ucgaggagcucacagucu	3	4	3
hsa-miR-204-3p	hsa-miR-204	gcugggaaggcaaagggacgu	1	7	6
hsa-miR-204-5p		uucccuuugucauccuaugccu	10	8	1
hsa-miR-21-3p	hsa-miR-21	caacaccagucgaugggcugu	6	9	5
hsa-miR-21-5p		uagcuuaucagacugauguuga	23	17	16
hsa-miR-29a-3p	hsa-miR-29	uagcaccaucugaaaucgguua	7	10	1
hsa-miR-29b-3p		uagcaccauuugaaaucaguguu	2	2	2
hsa-miR-29c-3p		uagcaccauuugaaaucgguua	1	1	2
hsa-miR-30a-5p	hsa-miR-30	uguaaacauccucgacuggaag	78	58	48
hsa-miR-30c-5p		uguaaacauccuacacucucag	6	10	4
hsa-miR-30d-5p		uguaaacauccccgacuggaag	36	21	23
hsa-miR-30e-5p		uguaaacauccuugacuggaag	34	18	16
hsa-miR-378c		acuggacuuggagucagaagagugg	11	15	14
hsa-miR-409-3p	hsa-miR-409	gaauguugcucggugaaccccu	10	3	12
hsa-miR-409-5p		agguuacccgagcaacuuugcau	1	4	2
hsa-miR-663a	hsa-miR-663	aggcggggcgccgcgggaccgc	43	22	26
hsa-miR-663b		gguggcccggccgugccugagg	1	5	1
hsa-miR-92a-3p	hsa-miR-92	uauugcacuugucccggccugu	42	34	38

hsa-miR-92b-3p		uauugcacucgucccggccucc	11	16	8
hsa-miR-99a-5p	hsa-miR-99	aacccguagauccgaucuugug	4	6	4
hsa-miR-99b-5p		cacccguagaaccgaccuugcg	31	36	30
hsa-miR-10a-5p	hsa-miR-10	uacccuguagauccgaauuugug	11	4	2
hsa-miR-10b-5p		uacccuguagaaccgaauuugug	16	3	5
hsa-miR-125a-5p	hsa-miR-125	ucccugagacccuuuaaccuguga	12	22	12
hsa-miR-125b-1-3p		acggguuaggcucuugggagcu	3	1	3
hsa-miR-125b-5p		ucccugagacccuaacuuguga	43	31	18
hsa-miR-126-3p	hsa-miR-126	ucguaccgugaguaauaaugcg	2	1	1
hsa-miR-126-5p		cauuauuacuuuugguacgcg	6	5	7
hsa-miR-100-5p	hsa-miR-100	aacccguagauccgaacuugug	23	34	9
hsa-miR-101-3p	hsa-miR-101	uacaguacugugauaacugaa	12	12	6
hsa-miR-103a-3p	hsa-miR-103	agcagcauuguacagggcuauga	20	8	12
hsa-miR-107	hsa-miR-107	agcagcauuguacagggcuauca	3	1	4
hsa-miR-124-3p	hsa-miR-124	uaaggcacgcggugaaugcc	42	21	24
hsa-miR-1254	hsa-miR-1254	agccuggaagcuggagccugcagu	2	4	2
hsa-miR-128-3p	hsa-miR-128	ucacagugaaccggucucuuu	24	21	14
hsa-miR-1285-5p	hsa-miR-1285	gaucucacuuuguugcccagg	10	6	10
hsa-miR-1290	hsa-miR-1290	uggauuuuuggaucaggga	33	30	29
hsa-miR-1291	hsa-miR-1291	uggcccugacugaagaccagcagu	13	15	20
hsa-miR-1303	hsa-miR-1303	uuuagagacggggucuugcucu	11	6	3
hsa-miR-1307-5p	hsa-miR-1307	ucgaccggaccucgaccggcu	1	5	2
hsa-miR-134-5p	hsa-miR-134	ugugacugguugaccagagggg	2	1	5
hsa-miR-138-5p	hsa-miR-138	agcugguguugugaaucaggccg	26	37	24
hsa-miR-139-5p	hsa-miR-139	ucuacagugcacgugucuccagu	2	4	1
hsa-miR-140-3p	hsa-miR-140	uaccacaggguagaaccacgg	13	4	6
hsa-miR-143-3p	hsa-miR-143	ugagaugaagcacuguagcuc	29	32	34
hsa-miR-144-3p	hsa-miR-144	uacaguauagaugauguacu	1	1	3
hsa-miR-1468-5p	hsa-miR-1468	cuccguuugccuguuucgcug	1	3	3
hsa-miR-148a-3p	hsa-miR-148	ucagugcacuacagaacuuugu	6	6	3
hsa-miR-153-3p	hsa-miR-153	uugcauagucacaaaagugauc	6	6	6
hsa-miR-16-5p	hsa-miR-16	uagcagcacguaaauauuggcg	16	22	10
hsa-miR-186-5p	hsa-miR-186	caaagaauucuccuuuugggcu	30	18	31
hsa-miR-1908-3p	hsa-miR-1908	ccggccgccggcuccgccccg	6	6	10
hsa-miR-191-5p	hsa-miR-191	caacggaaucccaaaagcagcug	63	68	55
hsa-miR-192-5p	hsa-miR-192	cugaccuaugaauugacagcc	3	2	4
hsa-miR-193a-5p	hsa-miR-193	ugggucuuugcgggcgagauga	2	2	2
hsa-miR-1972	hsa-miR-1972	ucaggccaggcacaguggcuca	34	30	24
hsa-miR-199a-5p	hsa-miR-199	cccaguguucagacuaccuguuc	1	1	1
hsa-miR-205-5p	hsa-miR-205	uccuucauuccaccggagucug	1	4	4
hsa-miR-212-5p	hsa-miR-212	accuuggcucuagacugcuuacu	2	1	2
hsa-miR-218-5p	hsa-miR-218	uugugcuugaucuaaccaugu	8	4	2
hsa-miR-221-3p	hsa-miR-221	agcuacauugucugcuggguuuc	12	3	2
hsa-miR-222-3p	hsa-miR-222	agcuacaucuggcuacugggu	3	4	6
hsa-miR-23b-3p	hsa-miR-23	aucacauugccagggauuacc	1	3	1
hsa-miR-25-3p	hsa-miR-25	cauugcacuugucucggucuga	2	1	3
hsa-miR-27b-3p	hsa-miR-27	uucacaguggcuaaguucugc	33	26	21
hsa-miR-28-3p	hsa-miR-28	cacuagauugugagcuccugga	17	10	16
hsa-miR-299-3p	hsa-miR-299	uaugugggaugguaaaccgcuu	15	3	6
hsa-miR-338-3p	hsa-miR-338	uccagcaucagugauuuuguug	6	8	5
hsa-miR-339-5p	hsa-miR-339	ucccuguccuccaggagcucacg	1	1	1
hsa-miR-3614-5p	hsa-miR-3614	ccacuuggaucugaaggcugccc	1	1	3
hsa-miR-3615	hsa-miR-3615	ucucucggcuccucgcggcuc	6	5	1
hsa-miR-3648	hsa-miR-3648	agccgcggggaucgccgaggg	36	30	42
hsa-miR-3653-3p	hsa-miR-3653	ccuccugaugauucuucuuc	1	1	1
hsa-miR-3687	hsa-miR-3687	cccggacaggcguucgugcgacgu	104	70	84
hsa-miR-370-3p	hsa-miR-370	gccugcuggggugggaaccuggu	23	21	31
hsa-miR-375	hsa-miR-375	uuuguucguucggcucgcguga	1	1	3
hsa-miR-424-3p	hsa-miR-424	caaaacgugaggcgcugcuau	28	42	22
hsa-miR-4449	hsa-miR-4449	cgucccggggcugcgcgaggca	11	7	4
hsa-miR-4454	hsa-miR-4449	ggauccgagucacggcacca	27	20	32
hsa-miR-4461	hsa-miR-4461	gauugagacuaguagggcuagg	1	1	1
hsa-miR-4488	hsa-miR-4488	agggggcgggcuccggcg	20	10	22
hsa-miR-4492	hsa-miR-4492	ggggcugggcgcgcgcc	2	4	2
hsa-miR-4508	hsa-miR-4508	gcggggcugggcgcgcg	7	7	14
hsa-miR-4516	hsa-miR-4516	gggagaagggucggggc	32	30	20

hsa-miR-4787-5p	hsa-miR-4787	gcgggggguggcggcggcauccc	14	8	17
hsa-miR-484	hsa-miR-484	ucaggcucaguccccucccgau	3	3	2
hsa-miR-5095	hsa-miR-5095	uuacaggcgugaaccaccgcg	1	1	6
hsa-miR-5096	hsa-miR-5096	guuucaccauguuggucaggc	44	57	41
hsa-miR-5191	hsa-miR-5191	aggauaggaagaaugaagugcu	4	3	2
hsa-miR-5585-3p	hsa-miR-5585	cugaauagcugggacuacaggu	14	15	19
hsa-miR-615-3p	hsa-miR-615	uccgagccugggucucccucuu	34	15	18
hsa-miR-3195	hsa-miR-3195	cgcgccgggcccggguu	36	26	46
hsa-miR-381-3p	hsa-miR-381	uauacaagggcaagcucucugu	5	4	5
hsa-miR-410-3p	hsa-miR-410	aauauaacacagauggccugu	4	4	6
hsa-miR-411-5p	hsa-miR-411	uaguagaccguauagcguacg	6	8	3
hsa-miR-769-5p	hsa-miR-769	ugagaccucuggguucugagcu	4	10	4
hsa-miR-8061	hsa-miR-8061	cuuagauuagaggauauuguu	2	1	2
hsa-miR-9-5p	hsa-miR-9	ucuuugguuaucuagcuguauga	60	51	33
hsa-miR-93-5p	hsa-miR-93	caaagugcuguucgugcagguag	7	9	4

Supplementary Table S2: KEGG partway ranking summary with detail information.

Term	Term ID	Total Genes	Union Target	miRNA in the Term	Score
Pathways in cancer	5200	325	241	88	2.286
Axon guidance	4360	129	102	82	1.833
Mapk signaling pathway	4010	272	187	88	1.817
Focal adhesion	4510	199	149	86	1.791
Neurotrophin_signaling_pathway	4722	127	96	83	1.579
Regulation_of_actin_cytoskeleton	4810	213	147	83	1.374
Olfactory_transduction	4740	388	20	52	1.373
Glioma	5214	65	50	80	1.334
Prostate_cancer	5215	89	67	81	1.271
Endocytosis	4144	201	142	80	1.265
Small_cell_lung_cancer	5222	84	66	81	1.26
Wnt_signaling_pathway	4310	150	115	81	1.257
Melanoma	5218	71	52	81	1.252
Tgf-beta_signaling_pathway	4350	84	64	75	1.226
Chronic_myeloid_leukemia	5220	73	58	77	1.188
Pancreatic_cancer	5212	70	59	77	1.161
Ubiquitin_mediated_proteolysis	4120	135	89	80	1.118
Erbb_signaling_pathway	4012	87	68	79	1.105
Dilated_cardiomyopathy	5414	90	58	77	1.077
Long-term_potentiation	4720	70	51	67	1.066
Oocyte_meiosis	4114	112	73	79	1.062
Insulin_signaling_pathway	4910	137	95	81	1.06
Hypertrophic_cardiomyopathy_(HCM)	5410	87	56	76	1.056
T_cell_receptor_signaling_pathway	4660	108	78	78	1.022
Adherens_junction	4520	73	59	82	0.997
Ecm-receptor_interaction	4512	84	56	71	0.989
Renal_cell_carcinoma	5211	70	58	80	0.973
Arrhythmogenic_right_ventricular_cardiomyopathy_(ARVC)	5412	74	53	73	0.922
Mtor_signaling_pathway	4150	52	40	78	0.906
Cell_cycle	4110	124	75	80	0.903
Calcium_signaling_pathway	4020	177	98	77	0.899
Non-small_cell_lung_cancer	5223	54	41	77	0.88
Melanogenesis	4916	101	62	76	0.872
P53_signaling_pathway	4115	68	46	72	0.862
Fc_gamma_r-mediated_phagocytosis	4666	94	64	75	0.851
Progesterone-mediated_oocyte_maturation	4914	86	52	77	0.846
Adipocytokine_signaling_pathway	4920	68	50	72	0.807
Protein_processing_in_endoplasmic_reticulum	4141	166	108	75	0.801
Amoebiasis	5146	105	64	71	0.791

Chagas_disease	5142	104	67	75	0.784
Colorectal_cancer	5210	62	49	72	0.78
Tight_junction	4530	132	81	81	0.776
Bacterial_invasion_of_epithelial_cells	5100	70	55	74	0.773
Bladder_cancer	5219	42	34	73	0.767
Gnrh_signaling_pathway	4912	101	61	76	0.764
Cytokine-cytokine_receptor_interaction	4060	275	132	79	0.762
Chemokine_signaling_pathway	4062	189	111	82	0.745
Amyotrophic_lateral_sclerosis_(ALS)	5014	54	39	70	0.734
Jak-stat_signaling_pathway	4630	155	88	78	0.731
Acute_myeloid_leukemia	5221	57	44	75	0.717
Vegf_signaling_pathway	4370	76	47	78	0.713
Gap_junction	4540	90	55	74	0.701
Type_ii_diabetes_mellitus	4930	47	30	67	0.672
Protein_digestion_and_absorption	4974	80	42	64	0.67
Long-term_depression	4730	70	42	72	0.662
Aldosterone-regulated_sodium_reabsorption	4960	42	28	67	0.658
Pancreatic_secretion	4972	103	48	78	0.653
Gastric_acid_secretion	4971	74	46	72	0.642
B_cell_receptor_signaling_pathway	4662	75	45	74	0.637
Toxoplasmosis	5145	132	76	77	0.634
Shigellosis	5131	61	46	73	0.632
Apoptosis	4210	88	58	72	0.631
Phosphatidylinositol_signaling_system	4070	78	51	69	0.627
Vascular_smooth_muscle_contraction	4270	126	67	77	0.626
Endometrial_cancer	5213	52	40	72	0.619
Salivary_secretion	4970	89	47	74	0.609
Fc_epsilon_ri_signaling_pathway	4664	79	48	72	0.585
Cell_adhesion_molecules_(CAMS)	4514	133	69	81	0.585
Leukocyte_transendothelial_migration	4670	116	70	77	0.585
Hepatitis_C	5160	134	75	75	0.543
Neuroactive_ligand-receptor_interaction	4080	318	119	78	0.54
Notch_signaling_pathway	4330	47	33	68	0.523
Lysosome	4142	121	60	66	0.516
Vasopressin-regulated_water_reabsorption	4962	44	33	65	0.513
Toll-like_receptor_signaling_pathway	4620	102	60	71	0.512
Thyroid_cancer	5216	29	22	60	0.511
Viral_myocarditis	5416	70	34	68	0.505
Basal_cell_carcinoma	5217	55	37	65	0.496
Rna_transport	3013	144	76	75	0.494
Epithelial_cell_signaling_in_helicobacter_pylori_infection	5120	68	44	68	0.493
Rna_degradation	3018	57	25	67	0.489
Natural_killer_cell_mediated_cytotoxicity	4650	140	55	73	0.457
Purine_metabolism	230	161	67	70	0.452
Glycosaminoglycan_biosynthesis_heparan_sulfate	534	26	19	50	0.443
Huntington's_disease	5016	183	76	71	0.443
Spliceosome	3040	127	60	71	0.442
Phagosome	4145	154	64	72	0.432
Inositol_phosphate_metabolism	562	57	33	61	0.431
Alzheimer's_disease	5010	168	73	72	0.419
Nod-like_receptor_signaling_pathway	4621	62	31	57	0.41
Glycerophospholipid_metabolism	564	79	43	58	0.408
Hedgehog_signaling_pathway	4340	56	37	61	0.401
Snare_interactions_in_vesicular_transport	4130	36	26	57	0.4
Vibrio_cholerae_infection	5110	54	32	60	0.392
Systemic_lupus_erythematosus	5322	136	28	52	0.389
Parkinson's_disease	5012	130	39	60	0.384
Pathogenic_escherichia_coli_infection	5130	56	31	60	0.378
Rig-i-like_receptor_signaling_pathway	4622	71	34	62	0.372

Cardiac_muscle_contraction	4260	77	33	65	0.363
Prion_diseases	5020	36	21	59	0.352
Oxidative_phosphorylation	190	132	32	46	0.341
Hematopoietic_cell_lineage	4640	88	38	54	0.322
O-glycan_biosynthesis	512	30	18	46	0.322
Leishmaniasis	5140	72	33	58	0.321
N-glycan_biosynthesis	510	49	25	56	0.32
Lysine_degradation	310	44	25	58	0.317
Carbohydrate_digestion_and_absorption	4973	43	20	62	0.313
Ppar_signaling_pathway	3320	70	31	57	0.308
Pyrimidine_metabolism	240	99	36	49	0.289
Basal_transcription_factors	3022	35	19	45	0.289
Glycosphingolipid_biosynthesis_lacto_and_neolacto_series	601	26	14	47	0.287
Peroxisome	4146	79	31	58	0.277
Malaria	5144	51	29	51	0.277
Sphingolipid_metabolism	600	40	20	48	0.267
Cysteine_and_methionine_metabolism	270	36	18	50	0.253
Glycerolipid_metabolism	561	49	22	46	0.249
Abc_transporters	2010	44	22	49	0.248
Complement_and_coagulation_cascades	4610	69	16	52	0.242
Type_i_diabetes_mellitus	4940	43	19	46	0.234
Selenoamino_acid_metabolism	450	26	13	52	0.229
Graft-versus-host_disease	5332	41	13	42	0.228
Ether_lipid_metabolism	565	35	15	43	0.227
Allograft_rejection	5330	37	14	39	0.223
Intestinal_immune_network_for_iga_production	4672	48	20	41	0.214
Alanine_aspartate_and_glutamate_metabolism	250	32	19	41	0.212
Antigen_processing_and_presentation	4612	76	22	42	0.21
Arginine_and_proline_metabolism	330	54	22	44	0.207
Maturity_onset_diabetes_of_the_young	4950	25	10	35	0.203
Nucleotide_excision_repair	3420	44	20	48	0.201
Starch_and_sucrose_metabolism	500	53	22	42	0.193
Autoimmune_thyroid_disease	5320	52	12	35	0.178
Glutathione_metabolism	480	50	20	41	0.175
Cytosolic_dna-sensing_pathway	4623	56	16	34	0.17
Glycolysis_gluconeogenesis	10	65	19	35	0.169
Regulation_of_autophagy	4140	34	12	41	0.167
Amino_sugar_and_nucleotide_sugar_metabolism	520	47	17	37	0.163
Citrate_cycle_(TCA_cycle)	20	31	16	40	0.16
Glycosylphosphatidylinositol(GPI)-anchor_biosynthesis	563	25	9	41	0.159
Primary_immunodeficiency	5340	35	14	37	0.158
Fatty_acid_metabolism	71	43	19	32	0.152
Steroid_hormone_biosynthesis	140	56	18	29	0.15
Collecting_duct_acid_secretion	4966	27	10	36	0.149
Galactose_metabolism	52	26	13	39	0.148
Retinol_metabolism	830	65	18	28	0.147
Valine_leucine_and_isoleucine_degradation	280	44	16	33	0.144
Metabolism_of_xenobiotics_by_cytochrome_p450	980	71	18	24	0.139
Aminoacyl-trna_biosynthesis	970	63	13	25	0.134
Arachidonic_acid_metabolism	590	57	9	32	0.128
Fructose_and_mannose_metabolism	51	34	15	33	0.126
Drug_metabolism_cytochrome_p450	982	73	18	22	0.125
Base_excision_repair	3410	33	10	32	0.12
Taste_transduction	4742	52	13	29	0.12
Staphylococcus_aureus_infection	5150	55	12	27	0.119
Rna_polymerase	3020	29	9	27	0.119
Proteasome	3050	44	13	28	0.114
Glycine_serine_and_threonine_metabolism	260	32	8	25	0.113
Butanoate_metabolism	650	30	11	26	0.111

Pyruvate_metabolism	620	41	12	28	0.108
Tyrosine_metabolism	350	41	8	24	0.099
Tryptophan_metabolism	380	42	14	25	0.098
Asthma	5310	30	7	23	0.088
Phototransduction	4744	29	9	24	0.088
Dna_replication	3030	36	12	19	0.074
Drug_metabolism_other_enzymes	983	52	14	12	0.074
Histidine_metabolism	340	29	8	15	0.071
Linoleic_acid_metabolism	591	29	4	14	0.066
Porphyrin_and_chlorophyll_metabolism	860	43	15	11	0.064
Propanoate_metabolism	640	32	8	18	0.064
Pentose_and_glucuronate_interconversions	40	31	12	9	0.059
Pentose_phosphate_pathway	30	26	7	14	0.053
Ascorbate_and_aldarate_metabolism	53	26	11	6	0.052
Homologous_recombination	3440	28	8	9	0.041
Ribosome	3010	90	2	5	0.022

Supplementary Table S3: List of primer sets used for qRT-PCR

A. Primers used to confirm miRNA expression

miRNA Name	miRNA specific 5’ primer	mRQ 3’ Primer
hsa-miR-4792	5′-CGGTAGCGCTCGCTGGC-3′	Provided with Mir-X™ miRNA qRT-PCR SYBR® Kit (Clontech Laboratories, Inc., Takara Bio, CA, US)
hsa-miR-6087	5′-TGAGGCGGGGGGGCGAGC-3′	
hsa-miR-320a	5′-AAAAGCTGGGTTGAGAGGGCGA-3′	
hsa-miR-7704	5′-CGGGGTCGGCGGCGACGTG-3′	
hsa-miR-181a-5p	5′-AACATTCAACGCTGTCGGTGAGT-3′	
hsa-let-7a-5p	5′-TGAGGTAGTAGGTTGTATAGTT-3′	
hsa-miR-124-3p	5′-TAAGGCACGCGGTGAATGCC-3′	
hsa-miR-26a-5p	5′-TTCAAGTAATCCAGGATAGGCT-3′	

B. Primers used to detect target molecule transcript (mRNA) expression

Gene	Forward Primer	Reverse Primer
CDK2	5′-CACCGAGACCTTAAACCTCAG-3′	5′-TCAGAATCTCCAGGGAATAGGG-3′
CDK4	5′-TTCCCATCAGCACAGTTCG-3′	5′-TCTACATGCTCAAACACCAGG-3′
CDK6	5′-GTCTGATTACCTGCTCCGC-3′	5′-ACACGATTACATAGCCTCTGC-3′
IGFR	5′-AGTTATCTCCGGTCTCTGAGG-3′	5′-TCTGTGGACGAACTTATTGGC-3′
TGFBR1	5′-GCCAAATATCCCAAACAGATGG-3′	5′-ATGCCTTCCTGTTGACTGAG-3′
AR	5′-AATCCCACATCCTGCTCAAG-3′	5′-AAGTCCACGCTCACCATG-3′
ITGB1	5′-TGTAAGGAGAAGGATGTTGACG-3′	5′-CAACCACACCAGCTACAATTG-3′
ITGB3	5′-CAAGTGTGAATGTGGCAGC-3′	5′-TTTTCGTCATGTAGGGCTCC-3′
AKT3	5′-AGACCGTTTGTGTTTTGTGATG-3′	5′-GAATGTAGATAGTCCAAGGCAGAG-3′
PIK3R3	5′-GAAAGTCGAGATGGAGAGCAG-3′	5′-AGTTCTGTCGAATAGGGCATC-3′
NRAS	5′-AATACATGAGGACAGGCGAAG-3′	5′-GTTTCCCACTAGCACCATAGG-3′
KRAS	5′-GAGGGAGATCCGACAATACAG-3′	5′-TCTCGAACTAATGTATAGAAGGCATC-3′
MAPK1	5′-AGATCTGTGACTTTGGCCTG-3′	5′-TCAATGGACTTGGTGTAGCC-3′
STAT3	5′-TTCTGGGCACAAACACAAAAG-3′	5′-TCAGTCACAATCAGGGAAGC-3′
GAPDH	5′-CTTTTAACTCTGGTAAAGTGG-3′	5′-TTTTGGCTCCCCCCTGCAAAT-3′

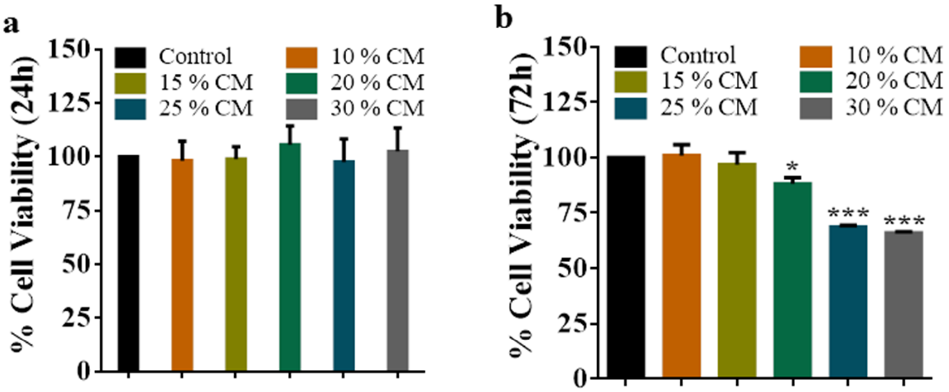

Supplementary Figure S1. Selection of hAMSC-CM dosage. a, % viability of A2780 cancer cells remained unchanged at 24 h of treatment with hAMSC-CM compared to control. **b,** at 72 h, cell viability started to decline upon 20 % CM incorporation; and optimum effect of CM was detected at 25% CM supplimentation. The experiments have been repeated at least three times. *p < 0.05, **p < 0.01 and ***p < 0.001.

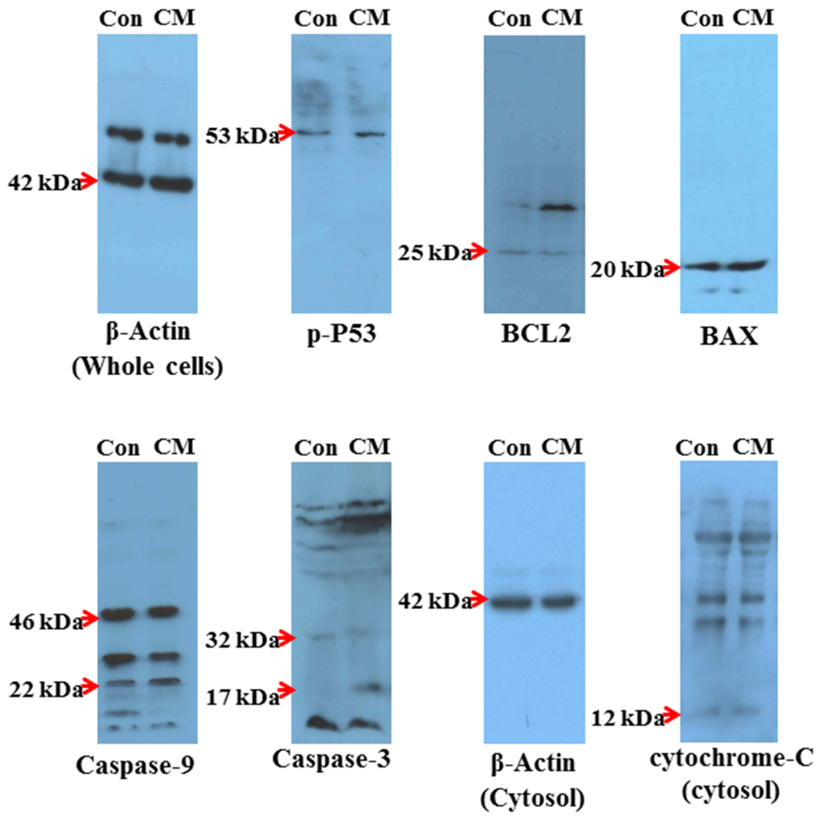

Supplementary Figure S2. Full length gel images of Figure-1f. The figure is showing the full length gel images of western-blot data presented in Figure-1f. The images are representative of three independent experiments.

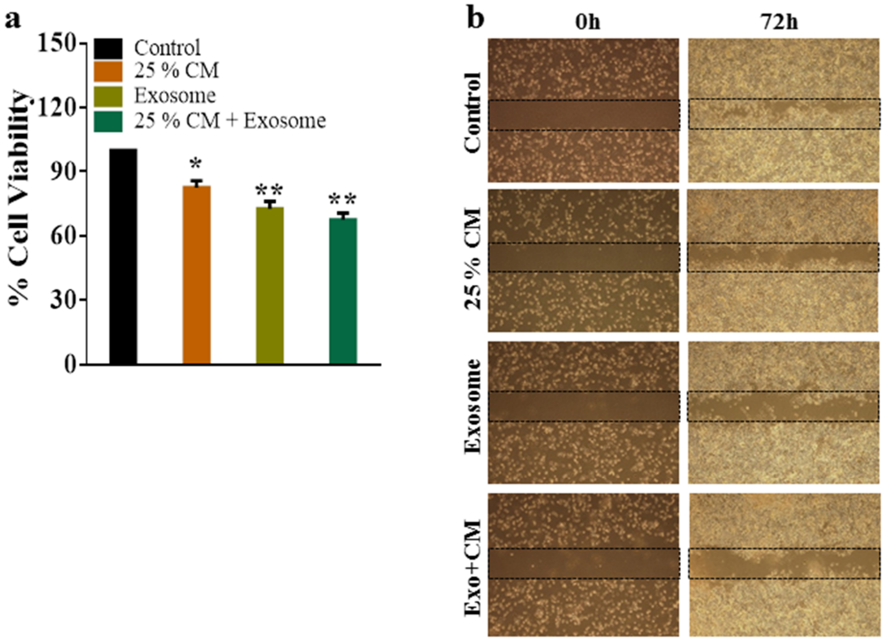

Supplementary Figure S3. Cell viability and wound healing assay using CM, exosomes or both CM and exosomes. a, % viability of A2780 cells at 72 h of treatment; internalization of exosomes reduced cell viability more efficiently than CM alone. **b,** exosomes internalization could restrain wound healing capacity of A2780 cells more effectively than CM alone. The experiments have been repeated at least three times. *p < 0.05 and **p < 0.01.

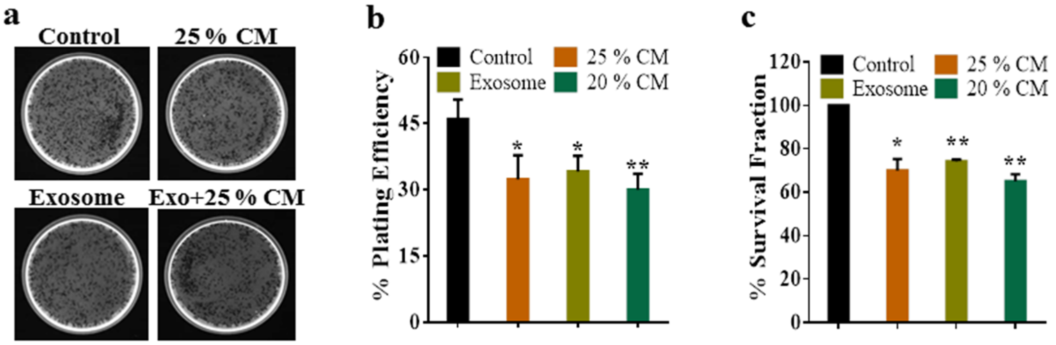

Supplementary Figure S4. Decreased colony formation of A2780 cells upon treatment with CM, exosomes or both CM and exosomes. a, colony formation under different conditions. **b,** % plating efficiency of A2780 cells reduced in all treated groups. **c,** % cell survival fraction decreased upon treatment with CM, exosomes or both CM and exosomes. The experiments have been repeated at least three times. $*p < 0.05$ and $**p < 0.01$.

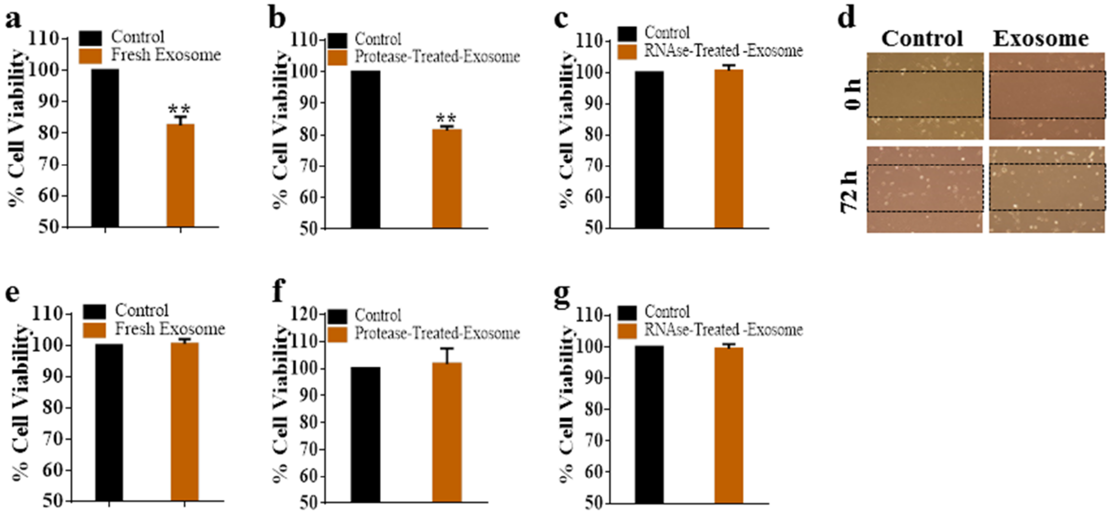

Supplementary Figure S5. hAMSC-CM-derived exosomes inhibited SKOV-3 cells, but could not inhibit CAOV-3 cells . a-d, internalization of exosomes inhibited the proliferation and wound-healing ability of SKOV-3 ovarian cancer cells. **e-g,** hAMSC-CM-derived exosomes could not inhibit the proliferation of CAOV-3 ovarian cancer cells. The experiments have been repeated at least three times. *$p < 0.05$ and **$p < 0.01$.

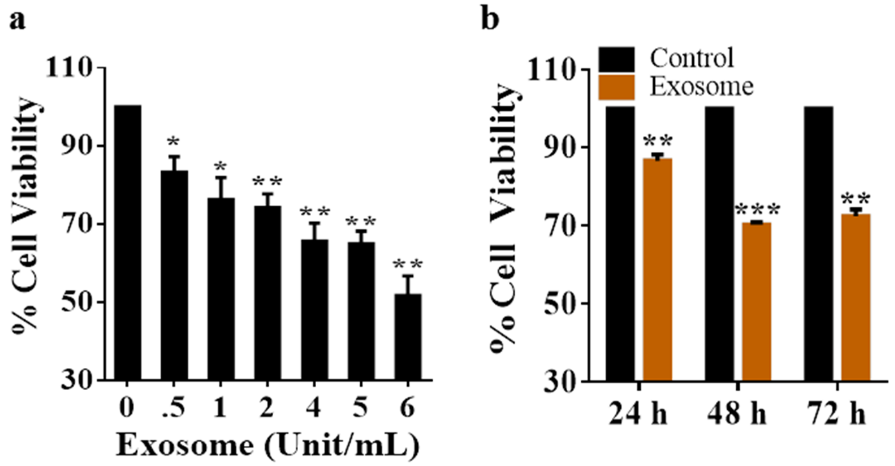

Supplementary Figure S6. hAMSC-CM-derived exosomes showed dose- and time-dependent inhibitory influence on A2780 ovarian cancer cells. a, the bar-diagram showing dose-dependent inhibitory influence of hAMSC-CM-derived exosomes on A2780 ovarian cancer cells. **b,** the data showing the time-dependent inhibitory effect of hAMSC-CM-derived exosomes on A2780 ovarian cancer cells. The experiments have been repeated at least three times. $*p < 0.05$, $**p < 0.01$ and $***p < 0.001$.

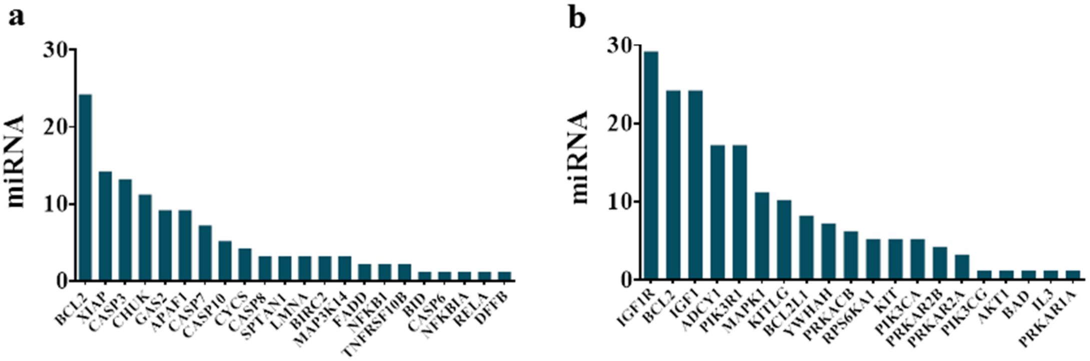

Supplementary Figure S7. *In silico* analysis of 'BioCarta signaling pathways'. a, molecules of **'**BioCarta death signaling pathway' predictably targeted by miRNAs detected in hAMSC-CM-derived exosomes. **b,** molecules belong to 'BioCarta BAD signaling pathway', potential targets for hAMSC-CM-derived exosomal miRNAs.

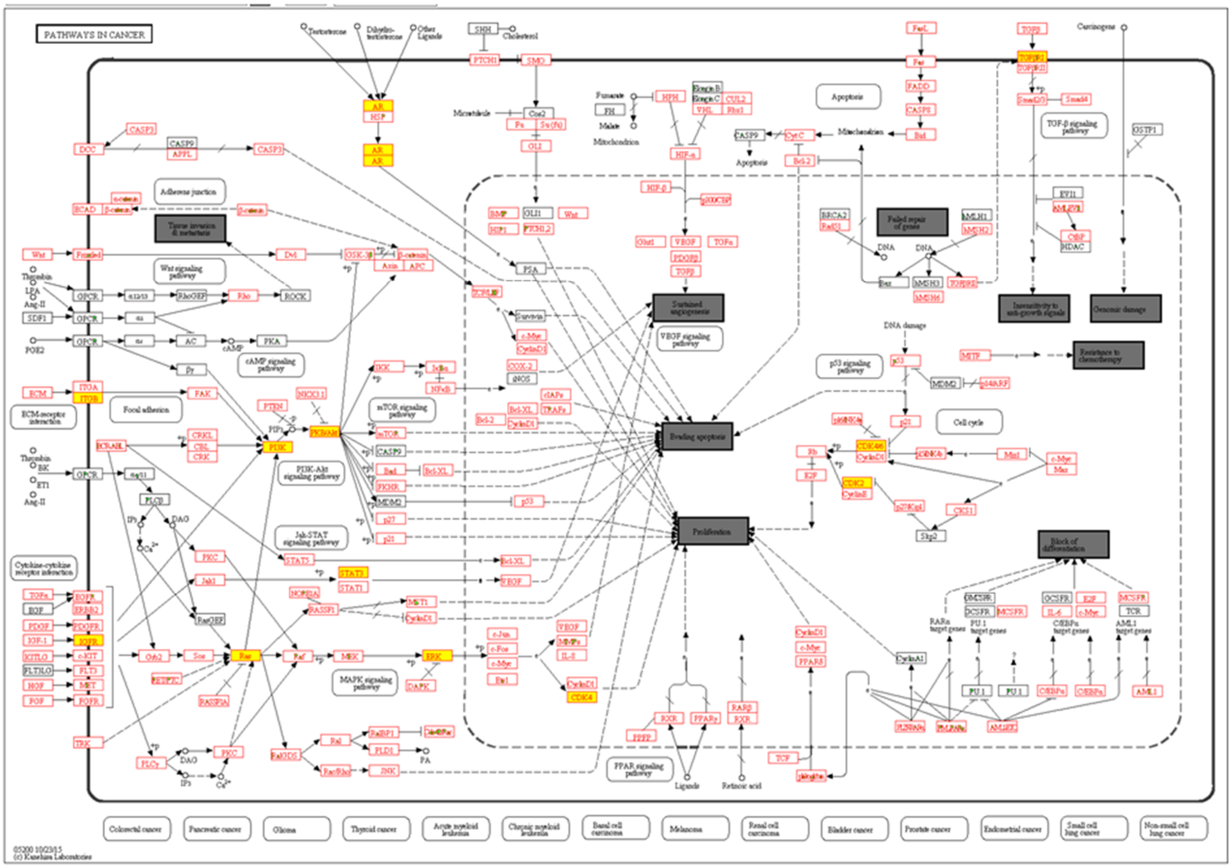

Supplementary Figure S8. KEGG 'Pathways in cancer'. Red colored molecules are predicted targets for 141 miRNAs detected in hAMSC-CM-derived exosomes. Yellow backgrounds represent the molecules, validated as miRNA targets by qRT-PCR. Kanehisa Laboratories (Kyoto University, Japan) kindly provided the KEGG pathway map image "Pathways in cancer (hsa05200)".

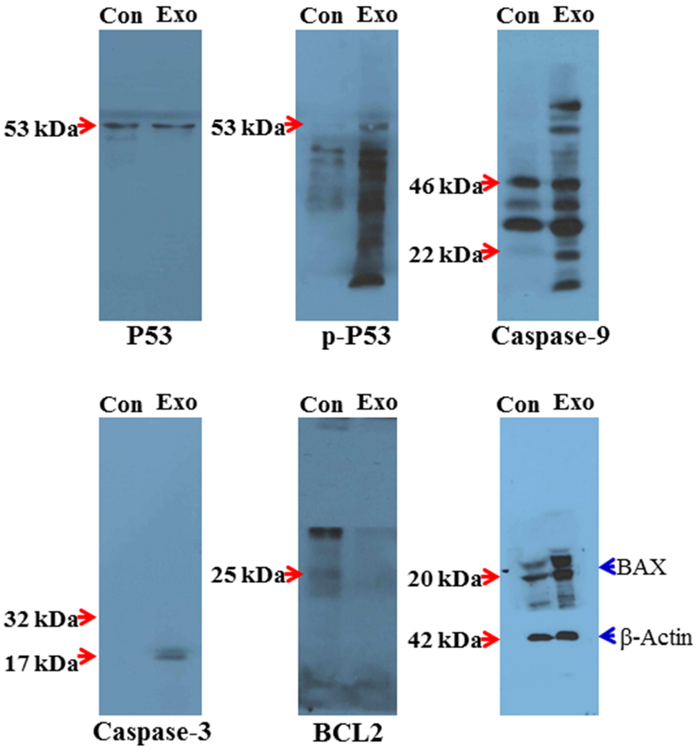

Supplementary Figure S9. Full length gel images of Figure-6a. The figure is showing the full length gel images of western-blot data presented in Figure-6a. The images are representative of three independent experiments.
